# Effects of music elements on mycophenolate glucuronidation in rat liver microsomes

**DOI:** 10.3389/jpps.2026.17260

**Published:** 2026-09-17

**Authors:** Jinal Adhiya, Ali H. Shalaby, Ala’A. R Al-Dajani, Sara R. El-Mahrouk, Carol Olympus, Heidi Ahonen, Ayman O. S. El-Kadi, Tony K. L. Kiang

**Affiliations:** 1 Faculty of Pharmacy and Pharmaceutical Sciences, University of Alberta, Edmonton, AB, Canada; 2 Faculty of Music, Department of Music Therapy, Wilfrid Laurier University, Waterloo, ON, Canada

**Keywords:** mycophenolic acid, glucuronidation, enzyme kinetics, music elements, tempo, rhythm, harmony, staccato

## Abstract

**Background:**

Mycophenolic acid (MPA), a first-line immunosuppressant for preventing transplant organ rejection, exhibits substantial inter- and intra-individual pharmacokinetic variability. We hypothesized that music elements (tempo, rhythm, and harmony) can affect the formation of MPA glucuronide (the major, inactive metabolite, MPAG) and MPA acyl glucuronide (the minor, toxic metabolite, AcMPAG) in Sprague Dawley (SD) rats.

**Methods:**

SD rats (8–10 weeks; 200–300 g; N = 8 per group [equal sex]) were exposed for 24 h in a sound-monitored room with music combinations (fast or slow tempo with regular or irregular rhythm) versus controls. Liver microsomes were analysed for MPAG and AcMPAG formation under initial velocity conditions. As FT IR (fast tempo with irregular rhythm) significantly reduced AcMPAG formation, we further tested harmony (tonal vs. atonal [AH]) within FT IR using three independent composers and two published pieces. MPA glucuronides were quantified by liquid chromatography–tandem mass spectrometry. Enzyme kinetics in FT IR AH versus controls were determined across MPA concentrations (0–320 μg/mL). mRNA and protein expression of the AcMPAG-forming enzyme (UDP-glucuronosyltransferase UGT2B1) and other expressed UGTs were analysed.

**Results:**

MPAG formation was unaffected by any music combination, whereas niflumic acid (100 μM, positive control) reduced it by ∼67%. AcMPAG formation was significantly reduced by FT IR (42.5 ± 14.4%; mean ± SEM; p < 0.05) and further reduced with added AH (74.4 ± 10.8%; p < 0.005). For MPAG, *V*_*max*_ and *CL*_*int*_ were comparable to controls, with a modestly higher *K*_*m*_. For AcMPAG, *V*_*max*_ and *CL*_*int*_ decreased by 89.7 ± 46.4% and 91.5 ± 29.7%, respectively (p < 0.05, n = 7 [3 males, 4 females]), with no change in *K*_*m*_. No sex differences in kinetic parameters were observed for either analyte. Inter-composer differences in MPA glucuronidation could be explained by the legato (continuous)-to-staccato (intermittent) articulation ratio. Hepatic *Ugt2b1* mRNA and protein expression were unchanged after FT IR AH exposure, as were hepatic *Ugt1a1, Ugt1a5, Ugt1a6, Ugt1a7,* and *Ugt2b12* mRNA.

**Conclusion:**

Our novel findings indicate that specific music elements (FT IR AH) reduced generation of the minor, toxic AcMPAG without affecting formation of the major, inactive MPAG. This is potentially therapeutically beneficial, as the adverse effects of MPA might be selectively attenuated without altering its clearance or therapeutic outcomes.

## Introduction

Mycophenolic acid (MPA) is a first-line immunosuppressant widely used in solid organ transplantation (i.e., kidney, heart, lung, heart-lung, liver) to prevent graft rejection [1–7]. It is administered as the ester prodrug mycophenolate mofetil (MMF) or the enteric-coated sodium (EC-MPA). MPA is commonly used in combination with calcineurin inhibitors and corticosteroids as part of the combinatorial immunosuppressive therapy [5, 8, 9]. Following administration, MMF and EC-MPA are rapidly converted to MPA, which inhibits lymphocyte proliferation by blocking the *de novo* guanosine nucleotide synthesis via inhibition of inosine monophosphate dehydrogenase (IMPDH) [1–9]. MPA has a high affinity for the IMPDH type II isoform, which is preferentially expressed in activated T and B lymphocytes, thereby mediating its immunosuppressive action [2, 10].

MPA is metabolized primarily to its major inactive MPA glucuronide metabolite (MPAG) and, to a lesser extent, to the pharmacologically active acyl glucuronide (AcMPAG), mainly via uridine diphosphate (UDP)-glucuronosyltransferases (UGT)1A9 and UGT2B7, respectively, in humans [11, 12]. Considerable interindividual variability in plasma concentrations has been reported [1–4, 6, 7], which can result in overexposure (increasing the risk of adverse effects) or underexposure (increasing the risk of acute organ rejection) [13–15]. Therefore, identifying clinical factors (i.e., in addition to age, sex, hemoglobin, race, albumin, body weight, creatine clearance, co-administered drugs, post-transplant time, and comorbidities [4, 5]) that contribute to this pharmacokinetic variability is essential to improve dosing individualization and optimize mycophenolate therapy.

Music has well-documented therapeutic benefits, including reducing stress and anxiety, supporting cardiovascular and respiratory function, alleviating pain, and altering physical function [16–18]. Specific music elements, such as tempo, rhythm, harmony, volume, melody, and timbre, can exert either stimulatory or inhibitory effects and may influence physiological responses in distinct ways [19]. In humans, variations in tempo (i.e., beats per minute), rhythm (i.e., the temporal pattern of sound), and harmony (i.e., the organization of tones and frequencies that contributes to perceived emotional variance) have been associated with changes in sympathetic and parasympathetic activities, heart rate, blood pressure, psychological and behavioural responses, cognitive function, and mood [20–22]. In rodents, music exposure has been reported to influence neurochemistry, physiology (e.g., sympathetic/parasympathetic nerve activity, blood pressure, corticosterone, prolactin, gastric emptying, and red blood cell deformity and aggregation), behaviour and learning, and immune modulators [23]. These effects may depend on both the type of music (e.g., classical, cultural, upbeat) and duration of exposure [23, 24]. Additionally, music and sound waves have been shown to alter sex hormone production (e.g., estrogen and testosterone) in rats [25, 26], potentially affecting biological function. Collectively, the same metabolism enzymes that regulate these hormones are known to metabolize xenobiotics (e.g., [27]), and music-associated changes in biological mediators (e.g., cytokines, cortisone) could potentially modify the expression and function of drug-metabolizing enzymes, suggesting that music could be an important extrinsic factor to influence the metabolism of drugs. Accordingly, investigating how specific music elements affects drug metabolism is potentially important for mitigating adverse drug effects and optimizing therapeutic benefits.

We hypothesized that music exposure is an influential factor that alters the intrinsic clearance of MPA. In this study, we evaluated a variety of music combinations, both original compositions by student composers and published music, systematically varying tempo (fast or slow), rhythm (regular or irregular), and harmony (tonal or atonal). We assessed their effects on MPA glucuronidation using liver microsomes isolated from male and female Sprague-Dawley (SD) rats following music exposure. The primary objective was to quantify music-related changes in the formation of the two pertinent MPA metabolites (MPAG and AcMPAG). Secondary objectives were to examine sex differences and to assess corresponding protein and gene expressions of the enzymes involved. To our knowledge, this is the first study to quantify how specific music elements modulate MPA metabolism.

## Materials and methods

### Chemicals and reagents

Rat UGT2B1 enzyme-linked immunosorbent assay (ELISA) kit was purchased from Abbexa LLC (cat# abx552486) (Sugar Land, TX, USA). The customized primers for real-time quantitative polymerase chain reaction (RT-qPCR) targeting the specific gene sequences were purchased from Integrated DNA Technologies, Inc. (Coralville, IA, USA). Acetic acid (cat#320099), acetonitrile (cat#34998-4L), alamethicin from *Trichodermia viride* (cat#A4665)*,* anhydrous sodium carbonate (cat# 791768-1 kg), anhydrous sodium chloride (cat#746398), bovine serum albumin (BSA, cat#A7906), cupric sulphate (cat# C1297-500g), Folin Ciocalteu’s phenol reagent (cat#F9252-500 mL), formic Acid (cat#F0507), high performance liquid chromatography (HPLC) grade methanol (cat#34860-4L-R), HPLC grade water (cat#270733), magnesium chloride (MgCl_2_; cat#M8266), MPA (cat#M5255), MPA-d_3_ solution (cat# M-137-1ML), protease inhibitor cocktail (PIC; mixture of 6 inhibitors i.e., AEBSF [4-(2-aminoethyl) benzenesulfonyl fluoride hydrochloride], aprotinin, bestatin hydrochloride, E-64 [N-(trans-epoxysuccinyl)-L-leucine 4-guanidinobutylamide], leupeptin hemisulfate salt, and pepstatin A; (cat# P8340)), sodium hydroxide (cat#S5881-500g), trisma hydrochloride (tris-HCL; cat#T5941), and uridine 5′-diphosphate glucuronic acid (UDPGA; cat#U6751) were acquired from Sigma-Aldrich (Oakville, ON, Canada). High-capacity complementary deoxyribonucleic acid (cDNA) reverse transcription kit (cat#4368814) and SYBR™ green polymerase chain reaction master mix (cat#4309155) were obtained from Applied Biosystems. D-sucrose (cat#BP220-1), TRIzol reagent (cat#15596018), phosphate-buffered saline (pH 7.4, cat#10010023), sodium potassium tartrate tetrahydrate (cat#S387-500), and ultrapure DNase/RNase-free distilled water (cat#10977-015) were purchased from Thermo Fisher Scientific (Waltham, MA, USA). MPA beta-D-glucuronide (MPAG; cat#TRC-M831520), MPA d3-beta-D-glucuronide (MPAG-d_3_; cat#TRC-M831522), and MPA acyl-beta-D-glucuronide (AcMPAG; cat#TRC-M831525) were obtained from Toronto Research Chemicals (Vaughan, ON, Canada).

### Animals

Adult Sprague Dawley (SD) rats (males: 130–150g; approximately 6 weeks old on arrival, females: 155–175 g; approximately 8 weeks old on arrival; with target weight range for both sex after acclimatization to be ∼200–300 g) [28] were obtained from Charles River Laboratories (Québec, QC, Canada). Prior to experiments, all animals were acclimatized for 14 days in the Health Sciences Laboratory Animal Services at the University of Alberta. A 12-h light/dark cycle was maintained, with *ad libitum* access to food (PicoLab rodent diet 20, Cat#5053, North American lab supply, Toronto, Canada) and water throughout acclimatization and experimental periods. Animals were housed in same-sex pairs in standard static cages without restraint. Music exposure studies were conducted in groups of eight rats (n = 4/sex). Following acclimatization, animals were placed in a room with monitored sound levels and continuously exposed in a continuous loop (for 24 h) to digital recordings of music delivered via digital speaker (JBL Charge 5, Harman International Industries, Northridge, CA, USA). Each group was exposed to combinations of two or three musical elements (i.e., tempo, rhythm, and harmony). Sound levels were monitored with digital decibel meters (axGear, Richmond, BC, Canada) positioned adjacent to cages, and maintained in the range from soft (60 ± 5 dB) to loud (80 ± 5 dB) [23]. At the conclusion of the music exposure period, animals were euthanized under isoflurane anesthesia (4% for induction, 2.5% for maintenance during organ collection) [29]. Livers were excised and stored at −80 °C until further analysis. The animal experiment was approved by the University of Alberta Research Ethics Board (animal use protocol no: AUP00004477; under the Animal Care and Use Committee; Health Sciences 1). For positive controls, SD rats (n = 1 for each sex) were treated with an intraperitoneal injection of 20 μg/kg 2,3,7,8-tetra chlorodibenzo-p-dioxin (TCDD) in corn oil (a known inducer for UGT enzymes [30]).

### Music combinations

To avoid inter-composer variability, we used music elements from a single student composer (Callum Drysdale; see Supplementary material for full bibliography) arranged in four tempo-rhythm combinations: fast tempo with regular rhythm (FT RR), slow tempo with regular rhythm (ST RR), slow tempo with irregular rhythm (ST IR), and fast tempo with irregular rhythm (FT IR). These musical pieces were utilized to assess their effects on MPA metabolism. Based on initial findings, two additional music combinations were created (by Callum Drysdale) by adding either atonal harmony (AH) or tonal harmony (TH) to the FT IR combination, which produced the strongest effects on MPA glucuronidation (see Results). Next, we compared music produced by three student composers (Callum Drysdale [original composer], Jasmine Hourahine, and Drew Aarssen; see Supplementary material for full bibliography) using the combination that showed the greatest effects (i.e., FT IR AH; see Results). We also tested two published works incorporating the same FT IR AH elements (i.e., First Construction (In Metal), by John Cage [31] and Violin Concerto Op. 36 - 3. Allegro, by Arnold Schoenberg [32]. The lawful copy of publicly available music files for these two published music pieces were played and exposed to animals for the purpose of academic publications, and therefore no copyright was required based on the assessments of University of Alberta Copyright Services Specialist. Digital audio files for our synthesized music can be provided upon request. Finally, to enable comparison across student-composed and published music with FT IR AH elements, we quantified the percentage of continuous (i.e., legato; [from Italian, meaning “tied” or “bound”] is a musical articulation indicating that notes should be played in a smooth, flowing, and connected manner—each note transitions seamlessly into the next with no silence between them [33]) and intermittent (i.e., staccato; [from Italian, meaning “detached”] is a musical articulation indicating that notes should be played in a short, sharp, and disconnected manner—each note is cut shorter than its written value, with a brief silence between notes [33]) frequencies from their individual spectrograms. To avoid potential confounding effects, the percentages of staccato and legato were kept constant (i.e., 99.67% and 0.33% respectively) in the initial screening between the FT IR, FT RR, ST IR, and ST RR groups.

### Microsomal isolation and protein quantification

Microsomal protein was isolated as previously described [34]. Briefly, 0.6 g of liver tissue was homogenized in 1.9 mL of homogenization buffer (5 μL PIC per mL of sucrose solution). The homogenate was centrifuged (Eppendorf centrifuge 5810 R 15-amp version, Hamburg, Germany) at 14,000 rpm for 20 min at 4 °C, and the resulting supernatant (i.e., S9 fraction) was ultracentrifuged (Beckman Coulter Optima MAX-XP ultracentrifuge, Mississauga, ON, Canada) at 40,000 rpm for 1 h at 4 °C. The microsomal pellet was resuspended in homogenization buffer by vortexing. Protein concentration was determined using the Lowry assay [35] with bovine serum albumin as the standard.

### Microsomal incubation conditions

Microsomal incubations were performed as previously described by our group [36]. Initial velocity conditions were established by varying microsomal protein concentration (0.05–1.05 mg/mL), incubation time (0–60 min), and MPA concentration (0.1–8 μg/mL [0.31–25 μM, MPA molecular weight: 320.34 g/mol]). Briefly, microsomal protein was preincubated with alamethicin (10 μg/mg protein) for 30 min. MPA was then added to a final reaction volume of 100 μL in incubation buffer (100 mM Tris-HCl, 10 mM MgCl_2_, and 1% BSA). The MPA stock solution (1,000 μg/mL, except for enzyme kinetic experiments where the high concentration stock was prepared as 50,000 μg/mL) was prepared in methanol, and the final methanol concentration in all incubations was maintained at <1% (vehicle concentrations in controls were matched to the exact methanol concentrations in the experimental groups). Reaction mixtures were equilibrated at 37 °C for 5 min in a shaking water bath (Precision shake bath model 25, Jouan Inc., Winchester, VA, USA). Reactions were initiated by adding UDPGA to a final concentration of 5 mM, and terminated by adding 152 μL of ice-cold protein precipitation solution containing internal standards (MPA-d_3_ [20 μg/mL, 1 μL] and MPAG-d_3_ [100 μg/mL, 1 μL]), together with 123 μL methanol, 25 μL acetonitrile, and 2 μL 10% acetic acid. MPAG and AcMPAG formation was quantified by liquid chromatography–tandem mass spectrometry (LC–MS/MS) as previously described [37] (see section *LC-MS/MS analysis for the quantification of MPA metabolites*). Specific incubation conditions for each experiment are provided in the corresponding figure and table legends.

### Enzyme kinetics of MPA glucuronidation in rat liver microsomes

The enzyme kinetic study was conducted in rat liver microsomes under initial velocity conditions using increasing concentrations of MPA (0–320 μg/mL, [0–998.9 μM, MPA molecular weight: 320.34 g/mol]) to determine kinetic parameters, including *V*_*max*_ (maximum reaction rate), *K*_*m*_ (substrate concentration at half *V*_*max*_), and intrinsic clearance (*CL*_*int*_; Equation 1). To determine the model of best fit, the concentration-velocity data (i.e., combined, male and female) were fitted in Graphpad Prism (version 10.5.0; GraphPad Software LLC, Boston, USA) to multiple kinetic models, including Michaelis-Menten, substrate inhibition, allosteric sigmoidal, and specific binding with hill slope. Model selection was based on visual inspection and goodness-of-fit statistics, as previously described [38], including R^2^ (coefficient of determination), sum of squares, root mean square, root mean square error, and the corrected Akaike information criterion [38]. Because only the Michaelis-Menten model (Equation 2) provided acceptable and best fits across all individual animals using these graphical and statistical criteria, it was used for subsequent analyses. Incubation conditions and the resulting *V*_*max*_, *K*_*m*_ *,* and *CL*_*int*_ values are provided in the corresponding figure and/or table legends.

*CL*_*int*_ was calculated using Equation 1 [38]:

$${CL}_{\mathrm{int}}=\frac{V_{\max}}{K_{m}}$$ (1)

Michaelis-Menten with Equation 2 [38, 39]:

$$v=\frac{V_{\max}\times \left[S\right]}{K_{m}+\left[S\right]}$$ (2)

Where, v is velocity, [S] is substrate (MPA) concentrations, *V*_*max*_, and *K*_*m*_ as defined above.

### LC-MS/MS analysis for the quantification of MPA metabolites

MPAG and AcMPAG formation in rat liver microsomal incubations was quantified using an LC-MS/MS assay developed in our laboratory [37] and partial-validated in this matrix in accordance with the current United States Food and Drug Administration bioanalytical method validation guidelines [40] (see supplementary material, Table 1). Briefly, after the incubation, 100 μL of reaction mixture (sample or calibrators) was mixed with 152 μL ice-cold protein precipitation solution containing 1 μL of MPA-d_3_ (20 μg/mL), 1 μL MPAG-d_3_ (100 μg/mL), 123 μL methanol, 25 μL acetonitrile, and 2 μL of 10% acetic acid. This mixture was vortexed-mixed for 30 s on a fixed speed vortex mixer (Fisher Scientific (Ottawa, Ontario, Canada), and centrifuged twice (Eppendorf centrifuge 5424 R, Hamburg, Germany) at 18,600 g at 4 °C for 10 min. A 10 μL aliquot of the resultant supernatant was injected for LC-MS/MS analysis. Chromatographic separation was performed on an Agilent Eclipse XDB C18 column (5 µm particle size, 4.6 × 250 mm diameter column) using mobile phases A and B (phase A: water with 2 mM ammonium acetate and 0.1% v/v formic acid, phase B: methanol with 2 mM ammonium acetate and 0.1% v/v formic acid). Gradient elution was used to achieve chromatographic separation (0–2 min: 30% phase B, 2–6 min: 30% → 100% phase B, 6–8 min: 100% phase B, 8–8.5 min 100% → 30% phase B, 8.5–15 min: 30% phase B). A 1 mL/min flowrate was maintained throughout the run time. Detection was achieved using a triple quadrupole mass spectrometer (LCMS-8050 Triple Quad LC-MS/MS; Shimadzu, Kyoto, Japan) [37].

**TABLE 1:** Forward and reverse primer sequences for real-time quantitative polymerase chain reaction (RT-qPCR).

Genes	Forward sequence	Reverse sequence	References
*Ugt1a1*	5’ -GCC​ATG​CAG​CCT​GGA​TTT-3′	5’ -CTC​TTG​GGC​ACG​TAG​GAC​AAC-3′	[41]
*Ugt1a5*	5’ -TCG​ACA​GTT​CTC​TTA​AGG​TCT​TGT​ATG-3′	5’ -AAG​GAG​CTG​GAA​TTC​AGA​TGC​T-3′	[41]
*Ugt1a6*	5′-CCG​CTA​TCG​CTC​CTT​TGG-3′	5’ -CTG​TAC​TCT​CTT​AGA​GGA​GCC​ATC​AG-3′	[41]
*Ugt1a7*	5′-CAG​ACC​CCG​GTG​ACT​ATG​ACA-3′	5’ -CAA​CGT​GAA​GTC​TGT​GCG​TAA​CA-3′	[41]
*Ugt2b1*	5’ -CTG​AAG​CAG​AGC​CCT​GAG​AGA-3′	5’ -GGG​AAG​GCA​CTG​GCA​TGA-3′	[41]
*Ugt2b12*	5’ -TGC​TGC​AAA​TAA​GTT​TCT​GCT​TTA​A-3′	5’ -TGA​CTA​TAT​TCC​ATC​GGC​CAT​ACC-3′	[41]
*β-actin*	5′-CCA​GAT​CAT​GTT​TGA​GAC​CTT​CAA-3′	5′-GTG​GTA​CGA​CCA​GAG​GCA​TAC​A-3′	[34]

### RNA extraction, cDNA synthesis and RT-qPCR

Total RNA was isolated from frozen liver tissue using TRIzol reagent (Invitrogen) as per manufacturer’s instructions (Invitrogen, Waltham, MA, USA). RNA extraction, quantification, and cDNA synthesis was performed as described in an earlier study [42, 43]. cDNA was amplified by RT-qPCR as described previously [44] using 96-well optical plates and the comparative delta delta cycle threshold (ΔΔCT) method [45] on a QuantStudio 3 system (Applied Biosystems, Thermo Fisher Scientific, Waltham, MA, USA). Forward and reverse primer nucleotide sequences (Table 1) for each gene were verified for the *Rattus norvegicus* using National Center for Biotechnology Information’s basic local alignment search tools (BLAST) through BLASTN 2.17.0+ web program version [46]. Fold changes in mRNA expression were considered statistically significant when the p-value was less than the Bonferroni-corrected alpha (α/k, where k = number of genes tested) [47]. Relative mRNA expression was calculated using β-actin as the housekeeping gene. mRNA fold change was calculated as 2^−Δ(ΔCT)^ using Equations 3, 4 [34]:

$$\Delta \text{CT}=\text{CT}\left(\text{target gene}\right)\;‐\text{ CT}\left(\beta ‐\text{actin}\right)$$ (3)

$$\Delta \left(\Delta \text{CT}\right)=\Delta \text{CT}\left(\text{FT IR AH}\right)\;‐\;\Delta \text{CT}\left(\text{control}\right)$$ (4)

### Enzyme-linked immunosorbent assay for rat UGT2B1 protein quantification

Protein expression of UGT2B1*,* an enzyme potentially involved in AcMPAG formation [11], was measured in liver microsomal protein from rats exposed to FT IR AH (composed by Callum) and from control rats. Sample dilutions were conducted as needed to ensure the samples were within the calibration range of the assay kit. Microsomal samples were processed according to the manufacturer’s instructions using a commercial enzyme-linked immunosorbent assay (ELISA) kit (Abbexa LLC; cat# abx552486; Sugar Land, TX, USA) to detect and quantify UGT2B1. Briefly, a 96-well plate pre-coated with an anti-UGT2B1 antibody was used. Microsomal protein (0.2 mg/mL; selected based on preliminary experiments) and standards (100 μL, 0.16–10 ng/mL, prepared from lyophilized protein) were incubated with pre-coated ELISA plate for 2 h at 37 °C, followed by incubation with detection reagent A (100 μL, detection antibody 100X (1:8,000) in 50% Glycerol) and B (100 μL, Horseradish peroxidase-conjugated Avidin 100X [1:30,000] in 50% Glycerol) for 1 h at 37 °C. After appropriate washing steps as per manufacturer’s instructions (wash buffer, tris buffer saline with 1% tween-20 and 0.33% Thymol), 0.05% 3,3′,5,5′-tetramethylbenzidine (90 μL, 10–20 min incubation) was used for colorimetric detection. Wells containing UGT2B1 produced a blue signal, which turned to yellow upon the addition of stop solution (50 μL, 1 M Sulfuric acid). Absorbance was measured immediately at 450 nm using a SpectraMax iD series multimode microplate reader (Molecular Devices LLC, San Jose, CA, USA). Relative absorbance was calculated by subtracting the matrix optical density, and UGT2B1 concentrations were determined from the standard curve.

### Statistical analysis

All results are presented as mean ± standard error of the mean (SEM). Statistical significance was set as *p* < 0.05; when multiple statistical tests were considered together, a Bonferroni-corrected alpha value was used. All analyses were performed in GraphPad Prism (version 10.5.0; GraphPad Software LLC, Boston, USA). Parametric tests (unpaired *t*-test or one-way analysis of variance (ANOVA) followed by Tukey’s *post hoc* test) were used when data met assumptions of normality and equal variance. When these assumptions were not met, nonparametric tests were applied (i.e., Mann–Whitney or Kruskal–Wallis ANOVA followed by Dunn’s test).

## Results

### Effects of tempo and rhythm

First experiments characterized the initial velocity conditions (i.e., linear reaction rates with MPAG or AcMPAG formations that are <10% of the initial incubated MPA concentrations [38] for microsomal protein (i.e., 0.2 mg/mL), incubation time (i.e., 15 min), and MPA concentration (i.e., 1.2 μg/mL) using select female and male rodents. Negative control incubations (i.e., MPA-free, UDPGA-free, and protein-free) showed no detectable MPA metabolites (data not shown). MPAG and AcMPAG formation (Figure 1) was quantified for the first four music conditions (FT RR, ST RR, ST IR and FT IR; composed by Callum). In the combined sample (i.e., male and females, n = 8), none of the music combinations affected MPAG formation. In contrast, FT IR significantly reduced AcMPAG formation by 42.5 ± 14.4% (mean ± SEM, p < 0.05, n = 8) compared with the no-music control group, and by 31.2 ± 5.5% (p < 0.05, n = 8) compared to FT RR (Figures 1A,B). When analyzed by sex, AcMPAG formation in female rodents was reduced in FT IR group by 52.6 ± 17.6% (p < 0.005, n = 4) versus control and by 33.6 ± 4.6% (p < 0.05, n = 4) versus FT RR (Figure 1D). In male rodents, AcMPAG formation only showed a trend toward a reduction (24.9 ± 12.1%, p > 0.05, n = 4) (Figure 1F). Additionally, ST RR decreased AcMPAG formation in females by 39.1 ± 8.3% (p < 0.05, n = 4) versus the control (Figure 1D), an effect not observed in males. In contrast, none of the music elements affected MPAG formation in female or male rodents (Figures 1C,E). To confirm that the *in-vitro* microsomal incubation conditions were suitable for MPAG attenuation (a negative finding in our experiments), MPAG formation was also evaluated in the presence of 100 μM niflumic acid, an inhibitor of human microsomal UGT1A9 [48] which is the primary enzyme responsible for MPAG formation [36] (as a potential inhibitor toward the rodent orthologs for MPA glucuronidation [11, 49, 50]). Niflumic acid reduced MPAG formation demonstrating positive inhibitory control (Supplementary material, Figure 1).

**FIGURE 1:**
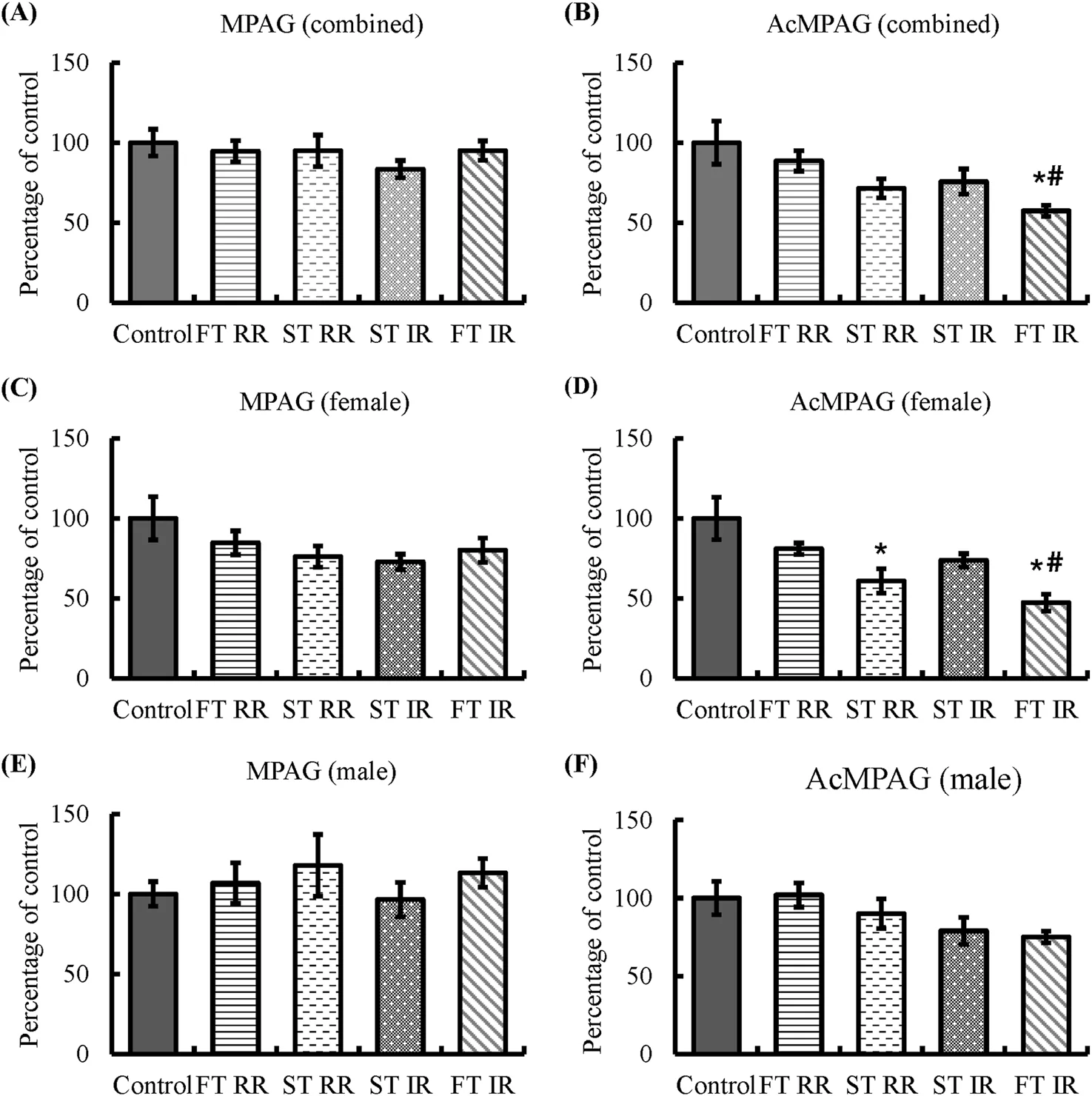
MPA glucuronidation in liver microsomes from Sprague Dawley rats incubated with 1.2 μg/mL MPA and 0.2 mg/mL protein for 15 min (initial velocity conditions). Methanol was maintained at 0.82% for all groups. Data are presented as mean ± SEM. FT: fast tempo, ST: slow tempo, RR: regular rhythm IR: irregular rhythm. **(A,B)** n = 8; 4 males, 4 females. *p < 0.05 vs. control (no music); #p < 0.05 vs. FT RR; Kruskal-Wallis test followed by Dunn’s multiple comparison test. **(C,D)** n = 4. *P < 0.05 vs. control (no music); #p < 0.05 vs. FT RR. One way ANOVA followed by Tukey’s test. **(E,F)** n = 4. P > 0.05 vs. control (no music).

### Fast tempo, irregular rhythm, with tonal or atonal harmony

Because the FT IR group showed a reduction in AcMPAG formation, we investigated this combination further by adding harmony (tonal or atonal) as an additional music element. We tested fast tempo and irregular rhythm with tonal (FT IR TH) or atonal harmony (FT IR AH) composed by Callum to assess their effects on MPAG and AcMPAG formations (Figure 2). MPAG formation was comparable to that in control animals in both the FT IR TH and FT IR AH groups (Figures 2A,C,E). In contrast, AcMPAG formation was reduced in the FT IR AH group by 74.4 ± 10.8 (p < 0.005, n = 8 combined sample), 71.2 ± 14.3% (p < 0.005, n = 4 [females]), and 80.1 ± 8.0% (p < 0.0005, n = 4 [males]) versus the control; and by 56.2 ± 11.7% (p < 0.05, n = 8), 64.5 ± 7.6% (p < 0.01, n = 4 [females]), and 41.5 ± 13.5% (p < 0.05, n = 4 [males]) versus the FT IR TH group (Figures 2B,D,F). Only the male rodents had a reduction in AcMPAG formation in the FT IR TH by 48.4 ± 13.4% (p < 0.05, n = 4) compared to the control (Figure 2F).

**FIGURE 2:**
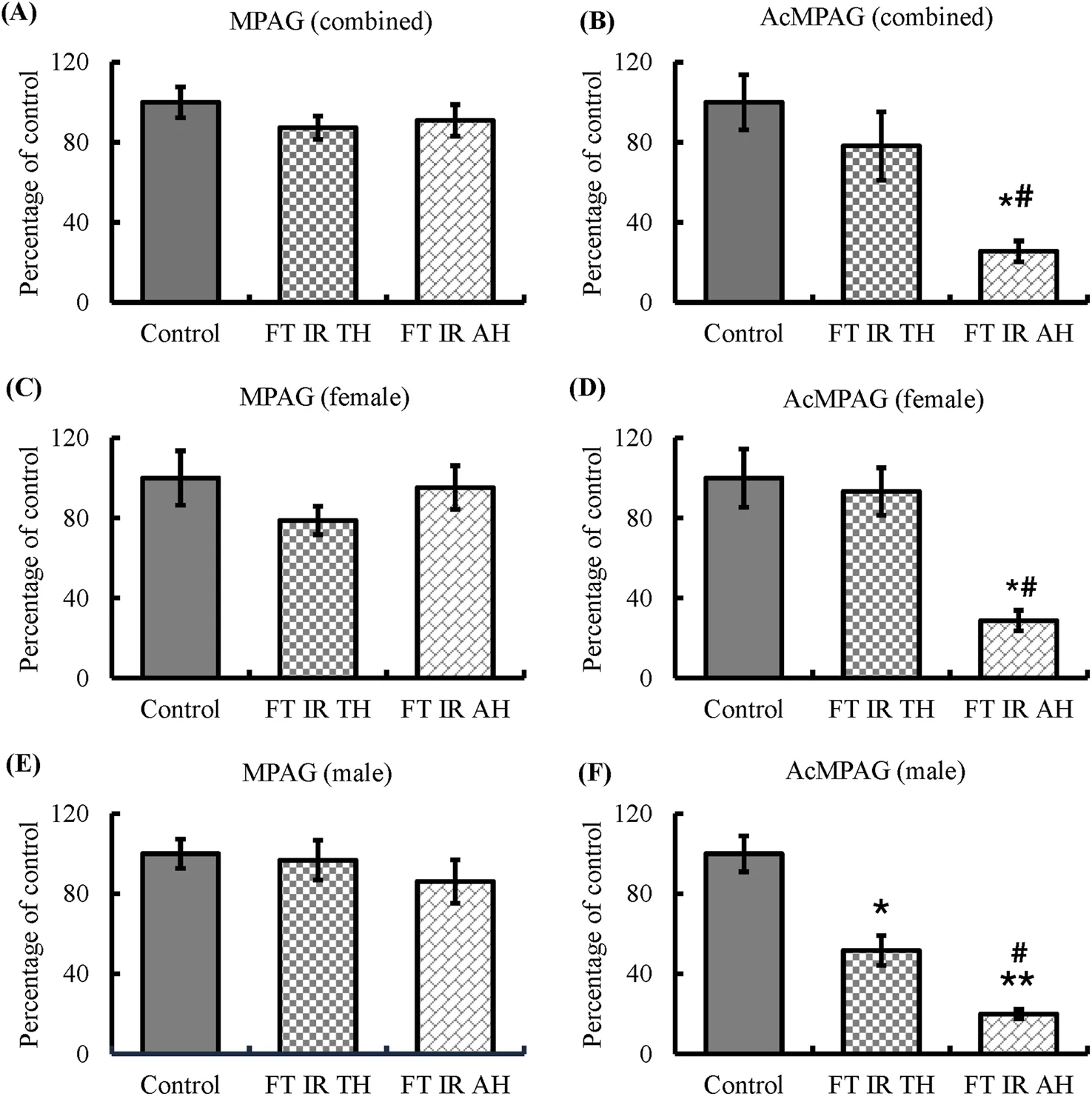
MPA glucuronidation in liver microsomes from Sprague Dawley rats incubated with 1.2 μg/mL MPA and 0.2 mg/mL protein for 15 min (initial velocity conditions). Methanol was maintained at 0.82% for all groups. Data are presented as mean ± SEM. FT IR TH: fast tempo, irregular rhythm, and tonal harmony; FT IR AH: fast tempo, irregular rhythm, and atonal harmony. **(A,B)** n = 8; 4 males, 4 females. *p < 0.005 vs. control (no music); #p < 0.05 vs. FT IR TH; one way ANOVA followed by Tukey’s test. **(C,D)** n = 4. *p < 0.005 vs. control (no music); #p < 0.05 vs. FT IR TH; one way ANOVA followed by Tukey’s test. **(E,F)** n = 4. *p < 0.05 vs. control (no music); **p < 0.0005 vs. control (no music); #p < 0.05 vs. FT IR TH; one way ANOVA followed by Tukey’s test.

### Enzyme kinetics of MPAG and AcPMAG formation

The effects of FT IR AH exposure on the enzyme kinetics of MPAG and AcMPAG formation are shown in Figure 3 for the combined sample (n = 8), while Table 2 summarizes sex-stratified kinetic parameters. Across all animals, enzyme kinetics were best described by the Michaelis-Menten model compared with alternative models. For MPAG formation, FT IR AH exposure was associated with a modest increase in *K*_*m*_ from 35.0 ± 2.84 μg/mL (controls, n = 8) to 49.9 ± 3.5 μg/mL (FT IR AH, p < 0.01, n = 8). Sex-stratified analyses showed only an increase in females (32.3 ± 10.7%, p < 0.05, n = 4, Table 2) in FT IR AH group versus the control group. In contrast, *V*_*max*_ and *CL*_*int*_ values for MPAG formation remained comparable to controls. For AcMPAG formation, *K*_*m*_ did not differ from controls; however, both *V*_*max*_ and *CL*_*int*_ were markedly reduced following FT IR AH exposure. *V*_*max*_ decreased by 89.7 ± 46.4% (p < 0.05, n = 7 [3 males, 4 females]), with only trends toward decreases observed in females (90.1 ± 72.3%, p > 0.05, n = 4) and males (89.1 ± 32.3%, p > 0.05, n = 3; Table 2). Similarly, *CL*_*int*_ decreased by 91.5 ± 29.7% (p < 0.05, n = 7 [3 males, 4 females]), including trends toward a reduction observed in females (94.0 ± 39.4%, p > 0.05, n = 4) and males (85.4 ± 24.8%, p < 0.05, n = 3, Table 2). In addition, there were no significant differences between male and female animals for *V*_*max*_ *, K*_*m*_ *, and CL*_*int*_ for MPAG and AcMPAG *within* the FT IR AH group. The lack of statistical significance in sex-stratified analyses was likely due to the small samples (i.e., secondary objectives).

**FIGURE 3:**
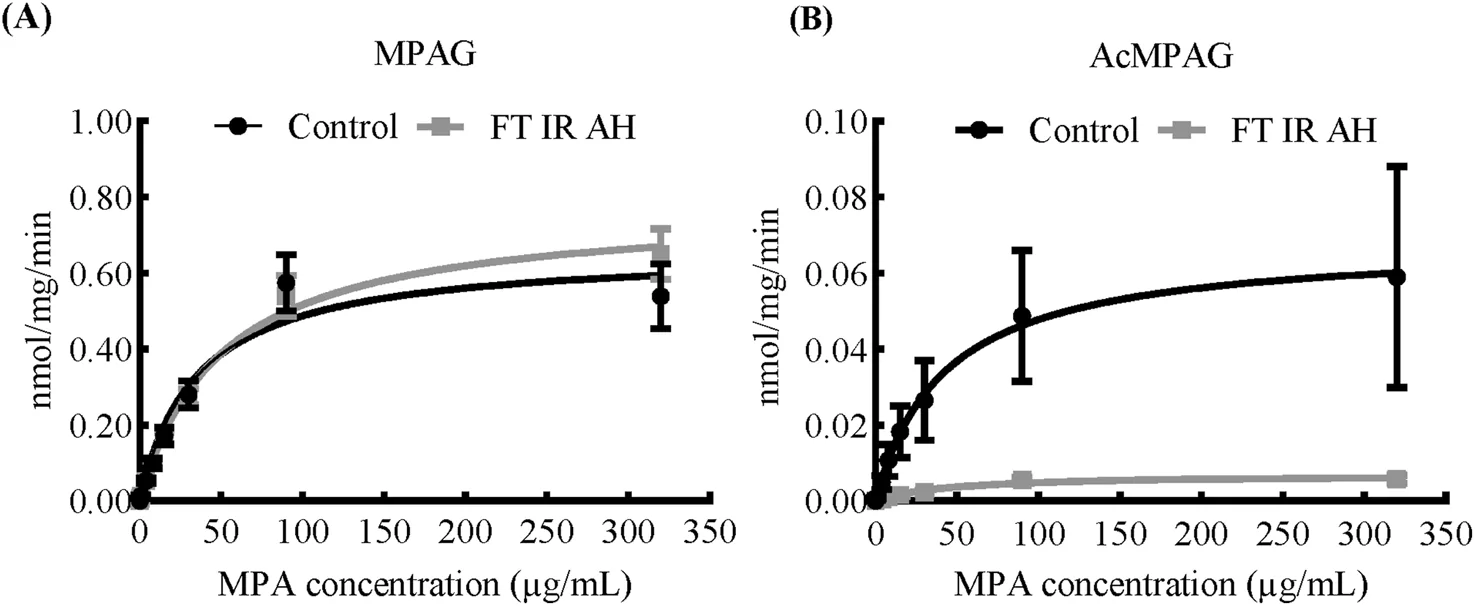
MPA **(A)** glucuronide **(B)** acyl glucuronide formation in liver microsomes from Sprague Dawley rats incubated with MPA (0, 0.1, 0.4, 0.8, 4, 8, 15, 30, 90, 320 μg/mL) and 0.2 mg/mL protein for 30 min (initial velocity conditions). Methanol was maintained at 0.66% for all groups. Data are presented as mean ± SEM (n = 8 [4 females, 4 males] MPA glucuronide; n = 7 [4 females, 3 males^a^] MPA acyl glucuronide) determined by GraphPad Prism 10 (Michaelis Menten fitting). MPA molecular weight: 320.34 g/mol used to convert between µg to nmol for the purpose of calculating intrinsic clearance (*CL*_*int*_ *= V*_*max*_ */K*_*m*_). Enzyme kinetic parameters for MPAG formation are *V*_*max*_:0.664 ± 0.100 nmol/mg/min, *K*_*m*_:35.0 ± 2.84 μg/mL, *CL*_*int*_:6.036 ± 0.708 μL/mg/min for control (no music) and *V*_*max*_:0.777 ± 0.0833 nmol/mg/min, *K*_*m*_:49.9 ± 3.54 μg/mL* (unpaired T-test), *CL*_*int*_:5.020 ± 0.440 μL/mg/min for fast tempo, irregular rhythm, and atonal harmony (FT IR AH). Enzyme kinetic parameters for AcMPAG formation are *V*_*max*_:0.0724 ± 0.0342 nmol/mg/min, *K*_*m*_:40.37 ± 10.64 μg/mL, *CL*_*int*_:0.594 ± 0.173 μL/mg/min for control (no music) and *V*_*max*_:0.00744 ± 0.00113 nmol/mg/min* (Mann-Whitney test), *K*_*m*_:56.8 ± 16.6 μg/mL, *CL*_*int*_:0.0504 ± 0.00890 μL/mg/min* (Mann-Whiteney test) for FT IR AH. *P < 0.05 vs. control (no music). ^a^MPA acyl glucuronide formation was below detection limit in one male rodent.

**TABLE 2:** Enzyme kinetics of MPAG and AcMPAG formation in liver microsomes from female and male Sprague-Dawley rats exposed to FT IR AH music.

Enzyme kinetic parameters	Control (no music)				FT IR AH (fast tempo, irregular rhythm, and atonal harmony)			
	MPAG		AcMPAG		MPAG		AcMPAG	
	Female (n = 4)	Male (n = 4)	Female (n = 4)	Male (n = 3)^a^	Female (n = 4)	Male (n = 4)	Female (n = 4)	Male (n = 3)^a^
*V*_*max*_ (nmol/mg/min)	0.673 ± 0.149	0.665 ± 0.157	0.0834 ± 0.0613	0.0577 ± 0.0217	0.660 ± 0.101	0.895 ± 0.113	0.00828 ± 0.00137	0.00632 ± 0.00234
*K*_*m*_ (µg/mL)	34.6 ± 2.95	35.4 ± 5.37	32.4 ± 12.5	50.9 ± 19.6	45.8 ± 2.85*	54.1 ± 6.22	74.1 ± 29.4	33.8± 1.78
*CL*_*int*_ ^b^ (µL/mg/min)	6.24 ± 1.29	5.83 ± 0.805	0.740 ± 0.289	0.400 ± 0.0978	4.72 ± 0.864	5.32 ± 0.311	0.0445 ± 0.00832	0.0583 ± 0.0190*

Rat liver microsomes were incubated with MPA (protein concentration = 0.2 mg/mL; incubation time = 30 min; MPA concentration = 0, 0.1, 0.4, 0.8, 4, 8, 15, 30, 90, 320 μg/mL) under initial velocity conditions. Data are presented as mean ± SEM (n = 8 [4 females, 4 males] for MPAG; n = 7 [4 females, 3 males^a^] for AcMPAG) and fitted in GraphPad Prism 10 using the Michaelis Menten model. *p < 0.05 vs. control (no music) using unpaired T-test.

^a^ AcMPAG formation was below detection limit in one male rodent.

^b^ MPA, molecular weight: 320.34 g/mol used to convert between µg to nmol for the purpose of calculating intrinsic clearance (*CL*_*int*_=*V*_*max*_/*K*_*m*_).

AcMPAG: MPA acyl glucuronide, *CL_int_*: intrinsic clearance; FT IR AH: fast tempo, irregular rhythm and atonal harmony; *K_m_*: concentration of substrate at which the reaction rate is half of the maximum; MPA: mycophenolic acid; MPAG: MPA glucuronide SD: Sprague-Dawley; SEM: standard error of mean; *V_max_*: maximum reaction rate.

### Comparison between three different composers of FT IR AH music on MPA glucuronidation

The effects of different composers’ FT IR AH music on MPAG and AcMPAG formations were further evaluated using music from Callum [C], Jasmine [J], and Drew [D] (Figure 4). MPAG formation was not affected by music from any composer (Figures 4A,C,E). In contrast, AcMPAG formation was reduced in rats exposed to music composed by Callum (by 72.8 ± 7.5%, p < 0.005, n = 8; 68.6 ± 13.0%, p < 0.005, n = 4 [females]; 77.3 ± 9.4%, p = 0.07, n = 4 [males]) and Jasmine (by 77.8 ± 8.4%, p < 0.0001, n = 8; 74.1 ± 13.6%, p < 0.005, n = 4 [females]; 81.8 ± 11.7%, p < 0.005, n = 4 [males]) compared with the control group. Music composed by Drew produced a smaller, non-significant reduction in AcMPAG formation (Figure 4). When directly compared with Drew’s music, AcMPAG formation was lower (by trend or statistical significance) in rats exposed to Callum’s music (by 35.3 ± 9.2%, p > 0.05, n = 8; 50.4 ± 14.1%, p < 0.05, n = 4 [females]; 19.4 ± 2.8%, p > 0.05, n = 4 [males]) and Jasmine’s music (by 40.3 ± 9.3%, p < 0.01, n = 8; 55.9 ± 13.8%, p < 0.01, n = 4 [females]; 23.9 ± 3.8%, p > 0.05, n = 4 [males]). The lack of statistical significance in sex-stratified analyses for male rodents was likely due to the small samples (i.e., a secondary objective).

**FIGURE 4:**
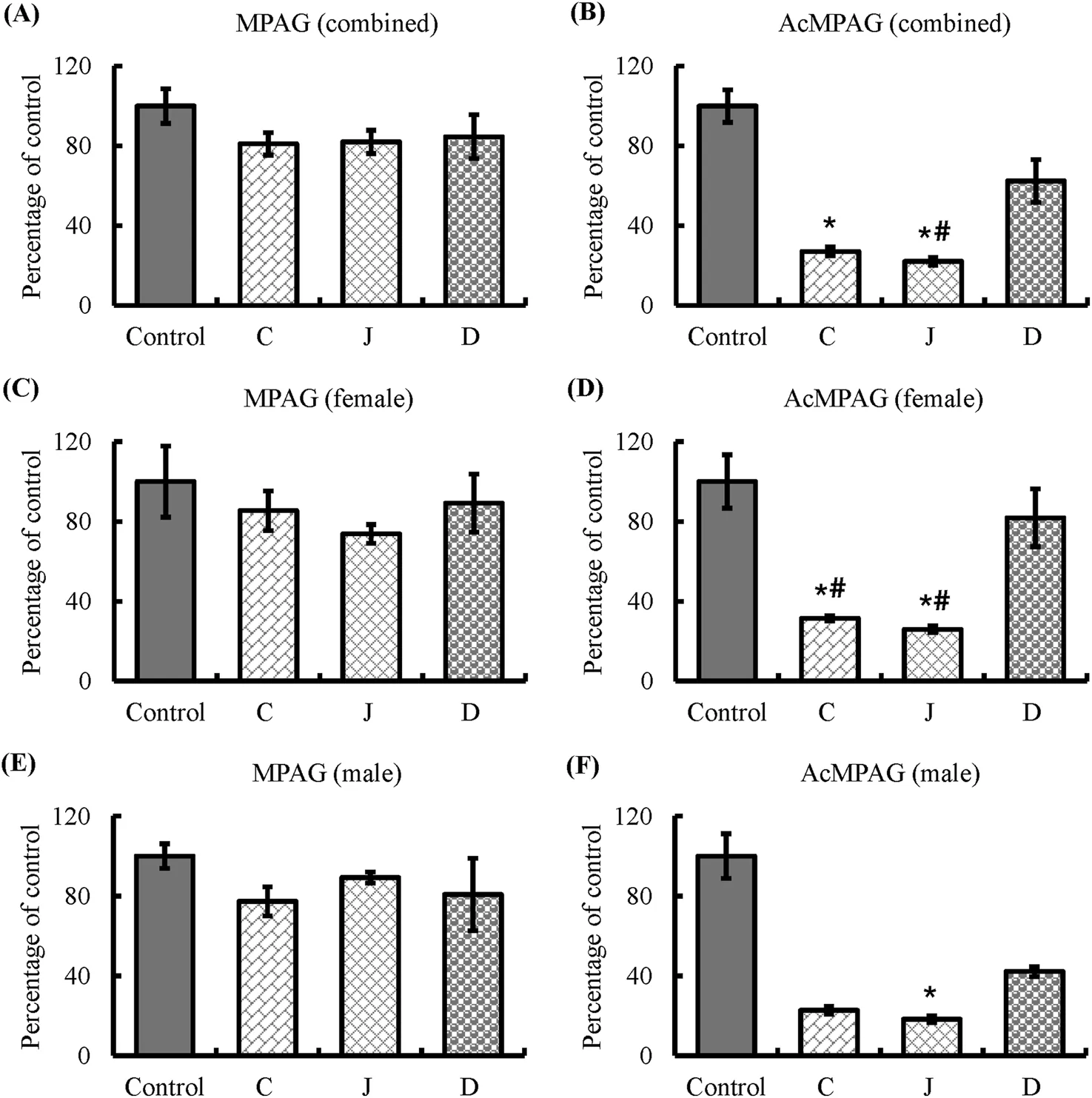
MPA glucuronidation in liver microsomes from Sprague Dawley rats incubated with 1.2 μg/mL MPA and 0.2 mg/mL protein for 15 min (initial velocity conditions). Methanol was maintained at 0.82% for all groups. Data are presented as mean ± SEM. Composers C: Callum, D: Drew; J: Jasmine. Music type: fast tempo, irregular rhythm, and atonal harmony. **(A,B)** n = 8; 4 males, 4 females. *p < 0.005 vs. control (no music), #p < 0.01 vs. Drew; Kruskal-Wallis test followed by Dunn’s multiple comparison test. **(C,D)** n = 4; *p < 0.005 vs. control (no music), #p < 0.05 vs. Drew; One way ANOVA followed by Tukey’s multiple comparison test. **(E,F)** n = 4; *p < 0.005 vs. control (no music); Kruskal-Wallis test followed by Dunn’s multiple comparison test.

### Effects of published, pre-composed music with FT IR AH on MPA glucuronidation

Rodents were also exposed to two published, pre-composed musical pieces [31, 32] with FT IR AH components to evaluate their effects on MPAG and AcMPAG formation (Figure 5). Consistent with all other mentioned music testing, MPAG formation was not affected by either of the music when compared to the controls. Moreover, AcMPAG formation in rats exposed to John cage music was also comparable to controls. In contrast, Arnold Schoenberg music reduced AcMPAG formation by 32.0 ± 10.7% (p < 0.05, n = 7 [4 males, 3 females]) versus the control. When analyzed by sex, a trend toward a reduction was observed in females (15.1 ± 13.2%, p > 0.05, n = 4) with a significant effect evident in males (45.6 ± 12.9%, p < 0.05, n = 4).

**FIGURE 5:**
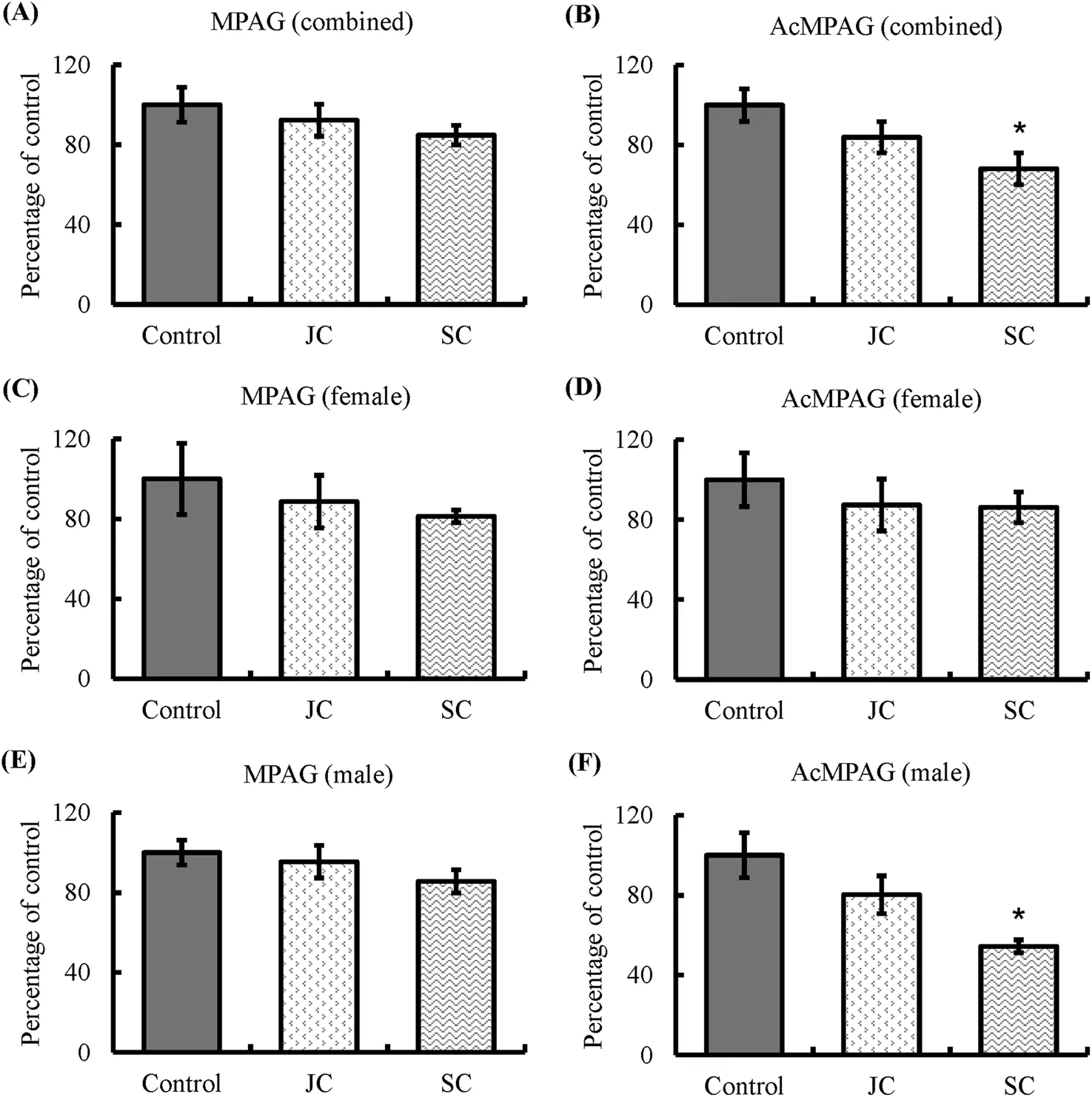
MPA glucuronidation in liver microsomes from Sprague Dawley rats incubated with 1.2 μg/mL MPA and 0.2 mg/mL protein for 15 min (initial velocity conditions). Methanol was maintained at 0.82% for all groups. Data are presented as mean ± SEM. Pre-composed music, JC: John Cage, SC: Arnold Schoenberg. Music type: fast tempo, irregular rhythm, and atonal harmony. **(A,B)** n = 7-8; 4 males, 4 females (note: n = 3 females for SC group), *p < 0.05 vs. control (no music), One way ANOVA followed by Tukey’s multiple comparison test. **(C,D)** n = 3. **(E,F)** n = 4, *p < 0.05 vs. control (no music); One way ANOVA followed by Tukey’s test.

### Analysis of music pieces with FT IR AH elements

In order to quantitatively delineate differences in the three student composers and the two published composers with FT IR AH elements (Figures 4, 5), the individual music pieces were analyzed for percentage of legato and staccato based on spectrograms (Supplementary material, Figure 3). The frequency range for all five music pieces were comparable within 20 Hz to 20 kHz (except Drew’s piece which had negligible frequency above 16 kHz and the whole piece represents intermittent breaks between the music frequencies). For Callum’s music, 68% of music exhibited tempo between 110 and 160 bpm, with the remaining 32% at ∼80 bpm, and the majority of the music was legato (i.e., 92.80% legato, 7.20% staccato). Jasmine maintained the tempo between 65 and 70 bpm also with high percentage of legato (92.52% legato, 7.48% staccato). In contrast, Drew maintained tempo at ∼105 bpm with intermittent presence of frequencies (i.e., 60.98% presence of audible frequencies and 39.02% gaps, where *virtually the whole piece* is composed as staccato above 900 Hz [and legato is present below 900 Hz, where SD rats hearing capacity starts at 250 Hz]) throughout the entire piece. John cage and Arnold Schoenberg maintained tempo between 90 and 126 bpm with reduced percentage of legato (i.e., John cage [18.24% legato, 81.76% staccato] and Arnold Schoenberg [22.16% legato, 77.84% staccato]).

### Effects of FT IR AH music on rodent hepatic uridine diphosphate (UDP)-glucuronosyltransferases (Ugt) mRNA expressions

mRNA expressions of rodent hepatic *Ugts* were measured in rats exposed to FT IR AH music (composed by Callum) and in control rats with no music exposure (Figure 6) using RT-qPCR. For each of the 6 *Ugt* genes examined, FT IR AH exposure produced changes of < 3-fold, and none was significant (i.e., using Bonferroni-corrected alpha value of 0.0083 based on 6 compared genes). For the *Ugt* enzymes potentially involved in MPA glucuronidation in rats (i.e., *Ugt1a1, 1a6, 1a7,* and *2b1*) [11, 49, 50], the overall fold changes were 1.24 ± 0.14, 1.60 ± 0.19, 1.37 ± 0.29, and 1.65 ± 0.36, respectively (p > 0.0083, n = 8 [4 males, 4 females]). When analyzed by sex, females showed fold changes of 1.18 ± 0.25, 1.59 ± 0.38, 0.95 ± 0.16, and 1.12 ± 0.36, respectively (p > 0.0083, n = 4), while males showed 1.31 ± 0.15, 1.62 ± 0.17, 1.79 ± 0.50, and 2.18 ± 0.53 fold increase respectively (p > 0.0083, n = 4). TCDD-exposed rats (i.e., positive control) showed substantial increases in *Ugt1a6* and *Ugt1a7* expression (∼34-fold and 14-fold, respectively n = 1 per sex) compared with controls (Supplementary material, Figure 2).

**FIGURE 6:**
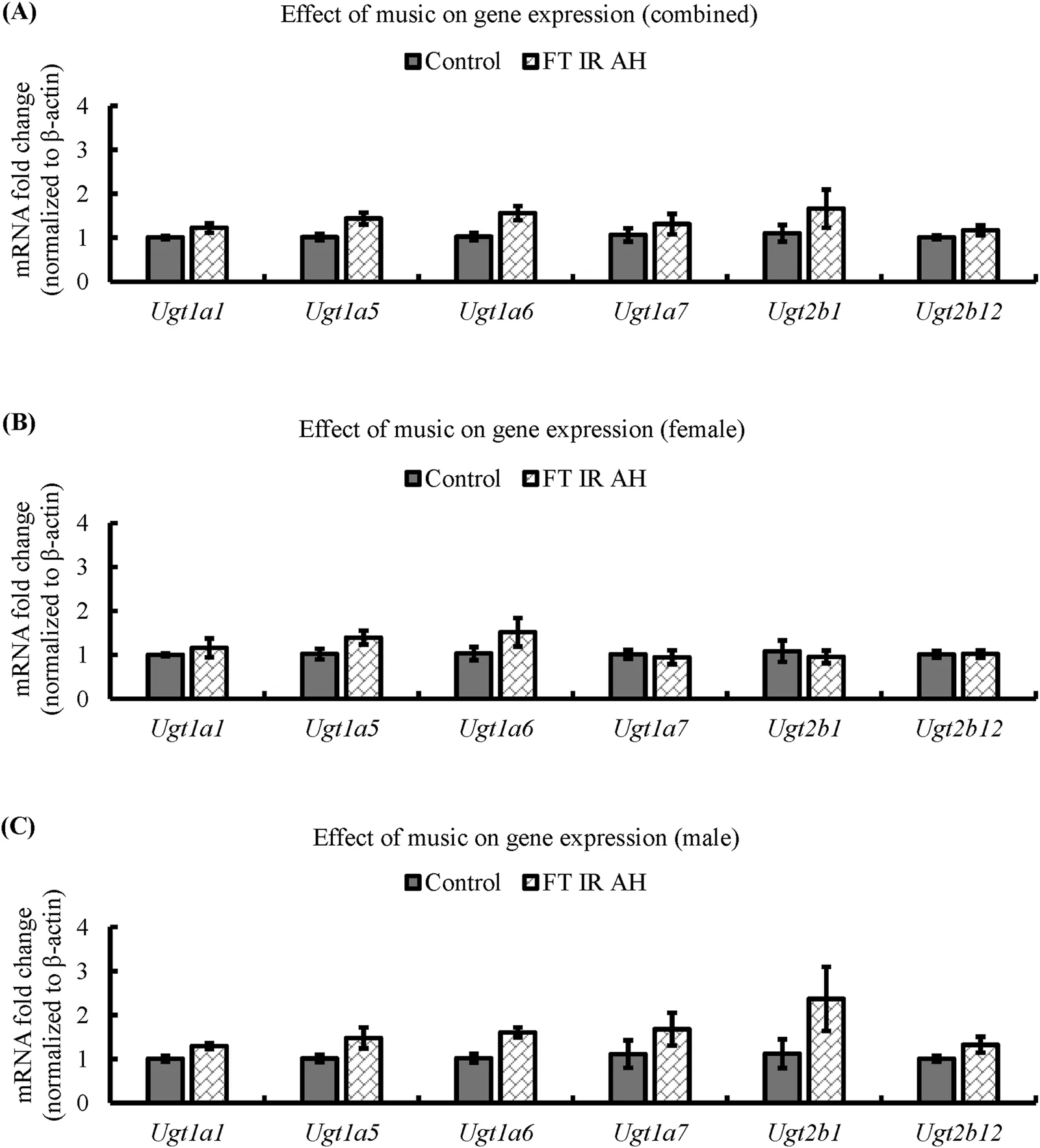
Effects of music (fast tempo, irregular rhythm, and atonal harmony) on mRNA expressions of rodent hepatic *Ugt* genes. Adult male and female Sprague Dawley rats were exposed to music for 24 h. After exposure, the liver was isolated and the mRNA expressions of *Ugt 1a1, 1a5, 1a6, 1a7, 2b1,* and *2b12* were determined by quantitative reverse transcription polymerase chain reaction. All data were normalized to beta actin. Results are represented as mean ± SEM, **(A)** n = 8 (4 males, 4 females); **(B)** females (n = 4); **(C)** males (n = 4). Data were analyzed using unpaired t-test or Mann Whiteny test compared to control animals (no music) with Bonferroni’s correction (i.e., alpha = 0.0083, based on 6 consecutive analyses). FT IR AH= fast tempo, irregular rhythm, and atonal harmony.

### Effects of FT IR AH music on rodent hepatic uridine diphosphate (UDP)-glucuronosyltransferases 2b1 protein expression

Notably, no differences in UGT2B1 protein expression, the putative enzyme for AcMPAG formation in rodents [11] were observed in the liver tissue of rodents exposed to control and the FT IR AH music (composed by Callum) (Figure 7). UGT2B1 protein concentrations in the FT IR AH group were 377.7 ± 29.6 ng/mL (p > 0.05, n = 8 [4 males, 4 females]), with similar values in females (387.8 ± 34.0 ng/mL, p > 0.05, n = 4) and males (367.7 ± 53.4 ng/mL, p > 0.05, n = 4) compared to the controls (368.8 ± 26.9 ng/mL [n = 8; 4 males, 4 females], 418.6 ± 36.2 ng/mL, [females, n = 4] and 319.1 ± 20.2 ng/mL [males, n = 4]).

**FIGURE 7:**
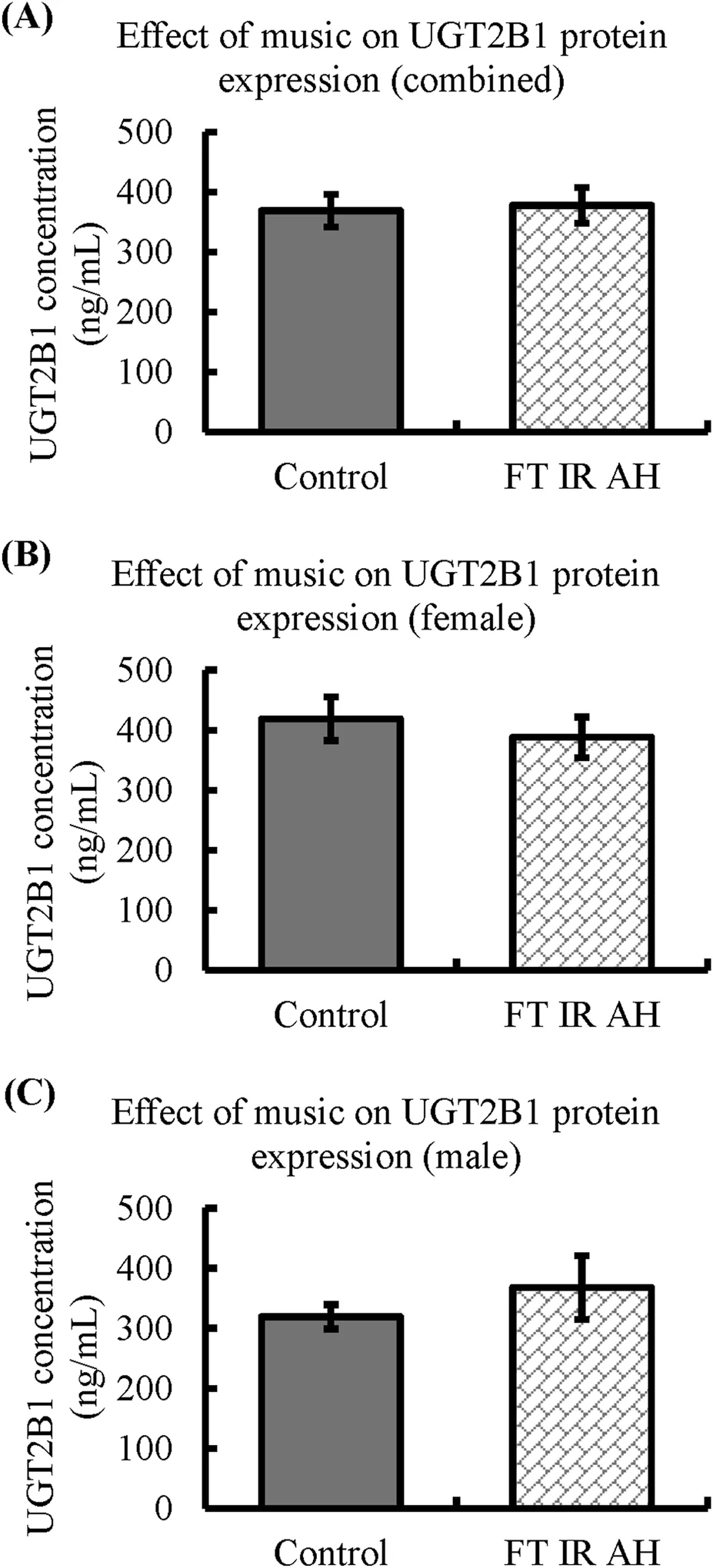
Effects of music (fast tempo, irregular rhythm, and atonal harmony) on protein expression of UGT2B1 in liver microsomes determined by enzyme-linked immunosorbent assay. Results are represented as mean ± SEM. **(A)** n = 8 (4 males, 4 females); **(B)** females (n = 4); **(C)** males (n = 4). Data were analyzed with unpaired t-test compared to control animals (no music). FT IR AH= fast tempo, irregular rhythm, and atonal harmony.

## Discussion

AcMPAG is a minor metabolite and is unlikely to contribute meaningfully to MPA’s therapeutic effects [4, 12] or overall drug clearance [1–4]. However, AcMPAG may be considered a toxic metabolite because it can stimulate the release of pro-inflammatory cytokines [51] and form protein adducts (based on rat studies) [52]. It has been linked to MPA toxicities in experimental models and in patients [1, 2]. Therefore, reducing AcMPAG formation through FT IR AH music exposure (Figure 3), a novel observation in our work, could be potentially therapeutically beneficial by decreasing side effects observed with mycophenolate therapy. In rats, UGT2B1 is the functional ortholog of human UGT2B7 and likely mediates AcMPAG formation [1, 11, 53]. Our enzyme kinetic analysis (Figure 3B) showed that AcMPAG formation was best described by the Michaelis–Menten model, consistent with Djebli et al. [54]. Although our *V*_*max*_ and *K*_*m*_ values in control animals were generally comparable to those reported previously [54], minor differences may reflect biological variability among individual animals, compared with pooled microsomes [54]. With FT IR AH music exposure, *K*_*m*_ remained similar to controls (Figure 3), suggesting that enzyme affinity or binding site conformations for MPA in the formation of AcMPAG was largely unchanged. In contrast, the substantial reduction in *V*_*max*_ observed in the FT IR AH group may be consistent with enzyme inhibition (e.g., [36, 38, 55]), post-translational modifications (e.g., [56, 57]), and/or reduced mRNA/protein expressions (e.g., [58, 59]). Notably, *Ugt2b1* mRNA expression and UGT2B1 protein levels in the FT IR AH group were comparable to controls (Figures 6, 7), suggesting that the reduction in AcMPAG *V*_*max*_ (hence *CL*_*int*_) may be due to these other possibilities. Therefore, additional studies are needed to determine whether the observed changes in enzyme activity are associated with preserved active site binding (i.e., through molecular docking or X-ray crystallography [57, 60], potential post-translational modifications (e.g., phosphorylation, ubiquitination, acylation [61]), and to identify factors that may alter catalytic efficiency from music exposure. For example, endogenous substrates of UGT enzymes, such as steroids, sex hormones, and bile acids, may also compete with MPA for UGT2B1 binding [62, 63]. Because music exposure can alter rodent neurochemistry, immune function, and physiological parameters [23], presumably through some of these mediators [64], it may be possible that music may also indirectly influence metabolism enzyme efficiency through these endogenous markers. Once identified, the precise mechanisms of inhibition (e.g., competitive, non-competitive, un-competitive) and inhibition potencies can be characterized directly in our experimental model (e.g., [36, 38]). Studies using different exposure durations, along with real-time monitoring of relevant biomarkers in an *in vivo* pharmacokinetic rodent model may also help identify the onset and the potential mediators for reduced AcMPAG formation and their associated pharmacokinetic perturbations.

MPAG is the major inactive metabolite of MPA and lacks immunosuppressive activity [1–4]. MPAG formation is mediated mainly by human hepatic UGT1A9 [1, 4, 11], whereas in rats, UGT1A1, UGT1A6, and UGT1A7 are likely key contributors [11, 49]. MPAG formations were not significantly different at experiments with single substrate concentrations (Figures 1, 2) but kinetic analysis with multiple substrate concentrations showed significant, but very modest, alteration in K_m_ values. Our kinetic analysis of MPAG formation in control animals followed Michaelis–Menten behavior (Figure 3A), consistent with *V*_*max*_ and *K*_*m*_ values observed in prior work [49, 54]. The lack of significant changes in *V*_*max*_ and *CL*_*int*_ values (with very modest increases in *K*_*m*_) (Figure 3) indicate no substantial influences by FT IR AH music (and in general no effects by all types of music tested in this work [Figures 1, 2]) on MPAG formation. This is supported by the lack of significant change in *Ugt1a1*, *Ugt1a6*, and *Ugt1a7* mRNA expressions (Figure 6) that suggest no influence of music on the regulation of metabolism enzymes likely responsible for the formation of MPAG. This observation may be therapeutically advantageous because MPA clearance (primarily through MPAG [1] might be expected to remain unchanged, which, in conjunction with our observation of reduced AcMPAG formation, would suggest that the FT IR AH music may be used strategically to *selectively attenuate* MPA toxicity (through AcMPAG reduction) without changing MPA therapeutic efficacy or clearance (through MPAG). However, due to the limitations of our *in vitro* hepatic microsomal model, we could not assess whether these music affected the other transporters (e.g., organic anion transporter 3, multidrug resistance-associated protein 2, and organic anion transporting polypeptide 1B1/3) which are known to affect the pharmacokinetics of MPAG [65, 66] (a potential future work, using an *in vivo* pharmacokinetic model described above with selective probes and physiologically-based pharmacokinetic modelling). Furthermore, FT IR AH music did not show a noticeable change in mRNA expression for other rat *Ugt* enzymes (*Ugt1a5,* and *Ugt2b12*) (Figure 6), pointing the potential beneficial effects of preserving these high-capacity, low-affinity metabolism pathways which are typically associated with xenobiotic detoxification [67].

We did not observe significant sex-dependent differences in the effects of music on MPAG or AcMPAG formation within the FT IR AH treatment group (Table 2), which may be confounded by the lack of sufficient sample size in this secondary sex-dependent analysis. Testosterone is a substrate of UGT2B1 in rodents [68], and sound exposure has been reported to increase testosterone production in male rat brain tissue [26]. Estradiol may also be a UGT2B1 substrate [69]. Other studies have reported altered serum estradiol, follicle-stimulating hormone, and luteinizing hormone concentrations in female rats following exposure to classical music [25]. Such hormonal shifts could modulate UGT2B1 transcription [70] and/or alter competition for enzyme binding, thereby affecting AcMPAG formation. Measuring circulating hormone concentrations during music exposure would help test this hypothesis and clarify sex-dependent effects on UGT2B1-mediated metabolism in a sufficiently powered cohort.

Spectrogram analyses of different composers’ FT IR AH music suggested that the percentages of staccato and legato may contribute to the differences observed in AcMPAG formation (Supplementary material, Figure 3). Callum and Jasmine’s compositions were more effective in reducing AcMPAG formation compared to Drew (Figure 4), potentially due to the higher staccato percentage in Drew’s music. Similarly, the published, pre-composed music by Cage and Schoenberg, with relatively higher staccato content, also produced a much smaller reduction in AcMPAG formation (Figure 5). Specific music elements and/or type of music are known to modulate the autonomic nervous system (i.e., heart rate, respiration rate and blood pressure) [71]. Specifically, staccato music is known to be associated with alteration in respiratory function (e.g., shorter inspiratory times, shorter expiratory times, larger minute ventilation [L/min, inspiratory volume/total breath duration]), and higher skin conductance [72]. On the other hand, legato music (in combination with slow tempo and minimal dynamic contrasts) is associated with reductions in respiratory rate, heart rate, and blood pressure [71]. Thus, it may be possible that the percentage legato vs. staccato in our tested music may have affected AcMPAG formation through sympathetic and parasympathetic alterations (e.g., altering hepatic blood flow and organ perfusion [71, 72]). Future investigations will include staccato/legato as a standalone music element.

A limitation of this study is that music exposure was restricted to a single 24-h period, which prevented us from determining the precise timings in the reduction of AcMPAG formation. This can be addressed with *in vivo* pharmacokinetic studies with frequent sampling (including MPA, MPAG, AcMPAG to estimate their individual metabolism clearance) with biomarker analysis (see below) and pharmacodynamic outcomes (e.g., neutropenia, acute rejection) to establish temporal and cause-effect relationships. Mechanistic experiments, including the analysis of additional blood biochemistry (e.g., sex hormones, inflammatory markers), could also help explain the pharmacodynamic changes associated with music exposure and, in conjunction with our pharmacokinetic study, clarify whether these changes are drivers or consequences for the reduction in AcMPAG formation. Moreover, the repeated 24-h exposure may create habituation, familiarity and/or changes in stress response over time which was not tested in this study. However, there were no visible signs of stress in animals during the music exposure and our testing of another enzymatic pathway using a similar experimental setup [43] did not report changes in mRNA expressions of IL-6. Further studies examining the effects of FT IR AH music on MPA immunosuppression, organ rejection, and adverse effects in a pre-clinical animal models, such as the heterotopic Lewis to Fisher rat cardiac transplantation [73], kidney allotransplantation (e.g., Dark Agouti–to-Lewis or Brown Norway–to-Lewis rats), heart allotransplantation (e.g., Dark Agouti–to-Lewis rats), aorta transplantation (e.g., Dark Agouti–to-Lewis rats), heart xenotransplantation (e.g., hamster-to-Athymic rnu/rnu rats or Lewis rats) [74], and the foetal rat pancreatic transplantation (e.g., Wistar rat to Wistar rats or Sprague-Dawley rats) [75]; which are well established animal models employing mycophenolate which may help translate our findings to the clinic. In addition, the observed effects on MPA metabolism may not be entirely attributed to the tested music elements (e.g., fast tempo, irregular rhythm and atonal harmony), as differences between each composer and the acoustic properties, as examples, would need to be examined systematically in the future. The music key and differences in instrumentation among music pieces need to be controlled in future studies as well.

In summary, our novel findings indicate that music with FT IR AH elements has differential effects on hepatic MPA intrinsic clearance and may represent an important clinical factor contributing to the variability in MPA pharmacokinetics and pharmacodynamics. Reductions in AcMPAG formation with this music combination (i.e., toxic metabolite) may be therapeutically beneficial to mitigate the side effects observed with MPA therapy without disturbing the major clearance pathway (i.e., major inactive metabolite, MPAG, formation). However, this potential novel observation requires further confirmation with *in vivo* pharmacokinetic studies and additional biomarker analyses.

## Data Availability

The original contributions presented in the study are included in the article/Supplementary Material, further inquiries can be directed to the corresponding author.
