# Supplementary material for: Effects of music elements on mycophenolate glucuronidation in rat liver microsomes
Source: J Pharm Pharm Sci. 2026 Sep 17;29:17260. doi: 10.3389/jpps.2026.17260 (PMC13627072; doi:10.3389/jpps.2026.17260)
Supplement: Supplementary file 1 [file Supplementaryfile1.pdf]

Supplementary information

Details of student composers:

Student composer 1: Callum Drysdale

Bachelor of Music in Integrated Musical Arts, Wilfrid Laurier University (Canada)

Student composer 2: Drew Aarssen

Bachelor of Music in Composition, Wilfrid Laurier University (Canada)

Student composer 3: Jasmine Hourahine

Bachelor of Music in Composition, Wilfrid Laurier University (Canada)

Supplementary figures

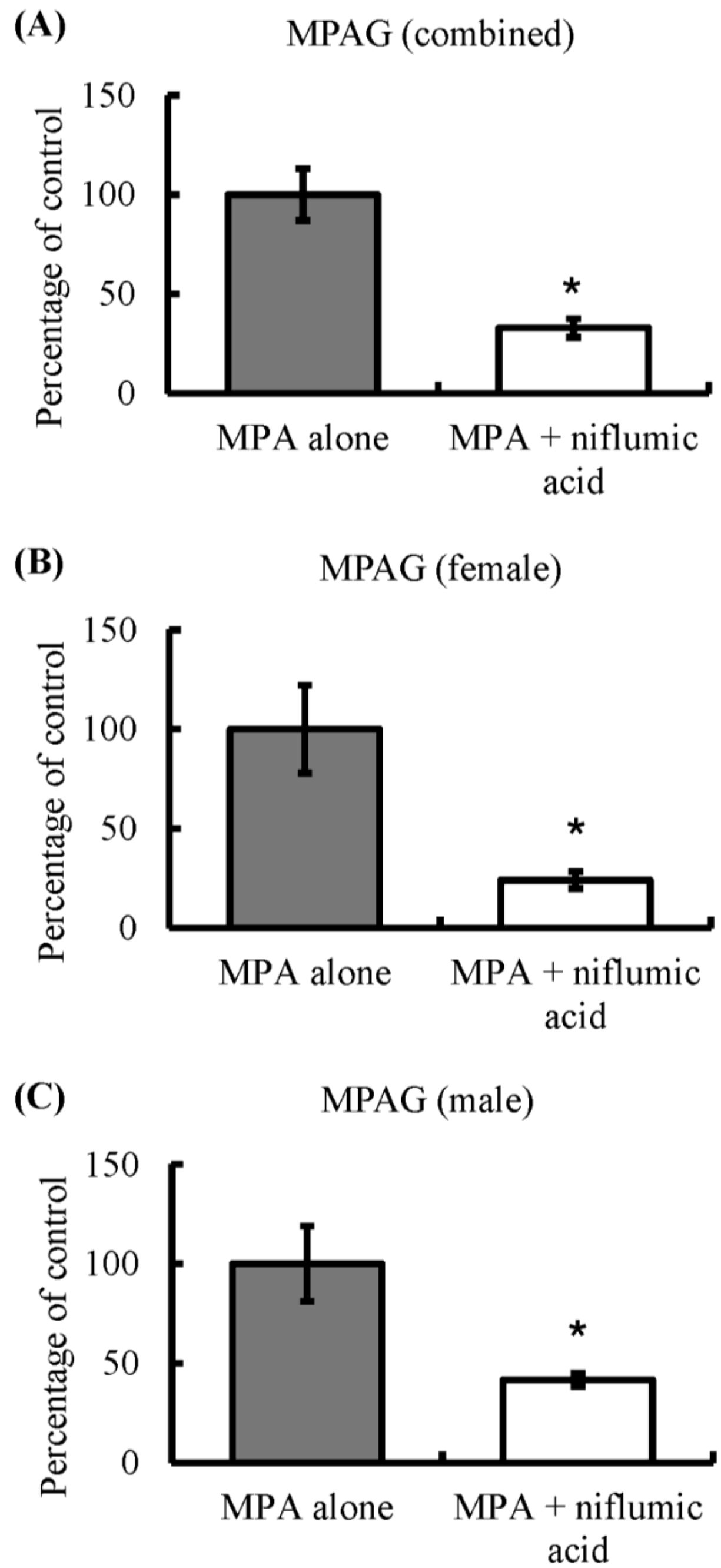

Figure 1. MPA glucuronide formation in liver microsomes from SD rats incubated with 1.2 µg/mL MPA and 0.2 mg/mL protein for 15 minutes (initial velocity conditions) with/without niflumic acid (100 µM). Methanol was maintained at 0.43% for all groups. **(A)** n=6; 3 males, 3 females; **(B)** n=3 females; and **(C)** n=3 males. Data are presented as mean ± SEM. *P<0.005 vs MPA alone using Mann-Whitney test or unpaired t test (two tailed).

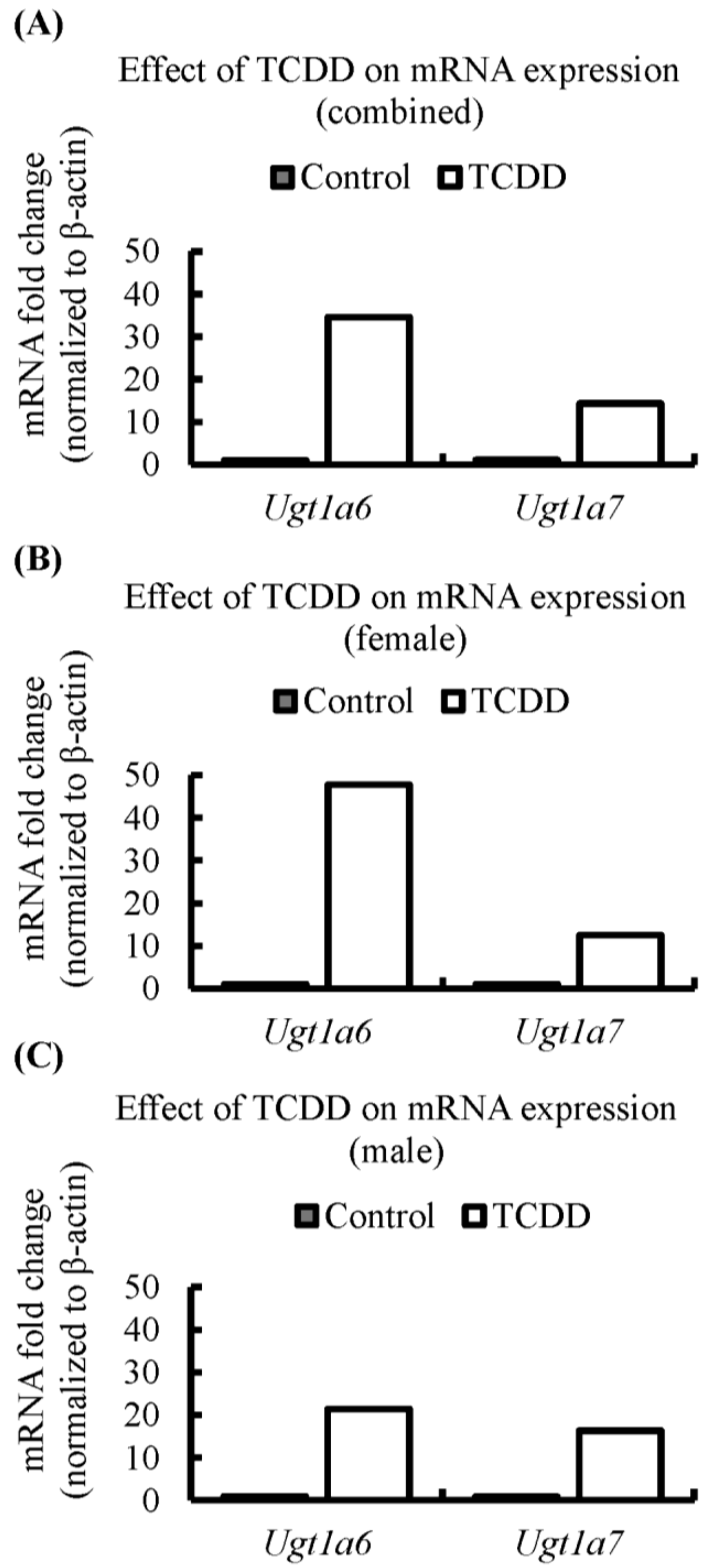

Figure 2. Effect of 2,3,7,8-Tetrachlorodibenzo-p-dioxin (TCDD) on mRNA expression. Sprague Dawley rats (n=1/sex) were exposed to 20 µg/kg TCDD in corn oil and livers were harvested post 24h exposure. mRNA expression was analysed in liver microsomal protein for UDP-glucuronosyltransferase (*Ug1a6, 1a7*). The results of all groups were normalized to the beta actin housekeeping gene. Results are represented as mean ± SEM, **(A)** n=8 control group (4 males, 4 females), n=2 TCDD group (1 male, 1 female) **(B)** females (n=4 for control group, n=1 for TCDD group) **(C)** males (n=4 for control group, n=1 for TCDD group).

(A)

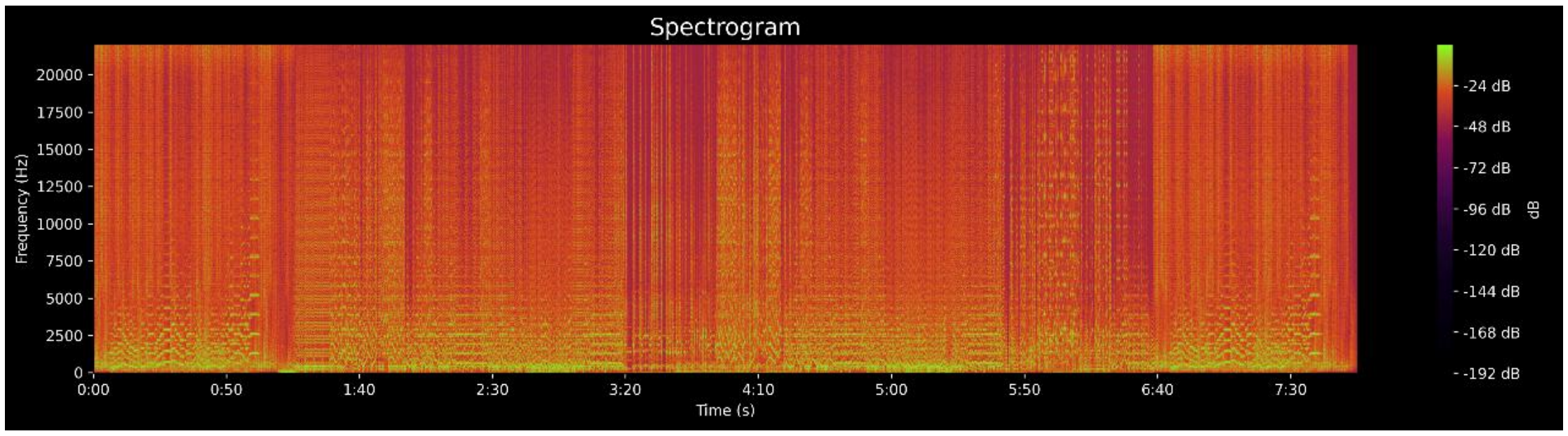
Spectrogram
Frequency (Hz)
0
2500
5000
7500
10000
12500
15000
17500
20000
Time (s)
0:00
0:50
1:40
2:30
3:20
4:10
5:00
5:50
6:40
7:30
dB
-24 dB
-48 dB
-72 dB
-96 dB
-120 dB
-144 dB
-168 dB
-192 dB

(B)

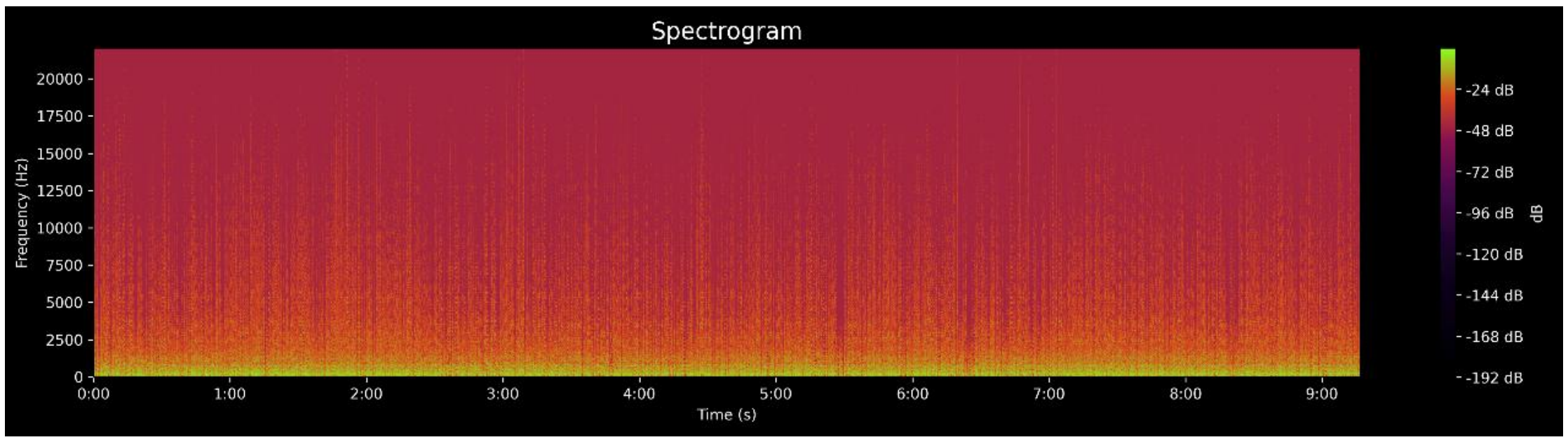
Spectrogram
Frequency (Hz)
0
2500
5000
7500
10000
12500
15000
17500
20000
Time (s)
0:00
1:00
2:00
3:00
4:00
5:00
6:00
7:00
8:00
9:00
dB
-24 dB
-48 dB
-72 dB
-96 dB
-120 dB
-144 dB
-168 dB
-192 dB

(C)

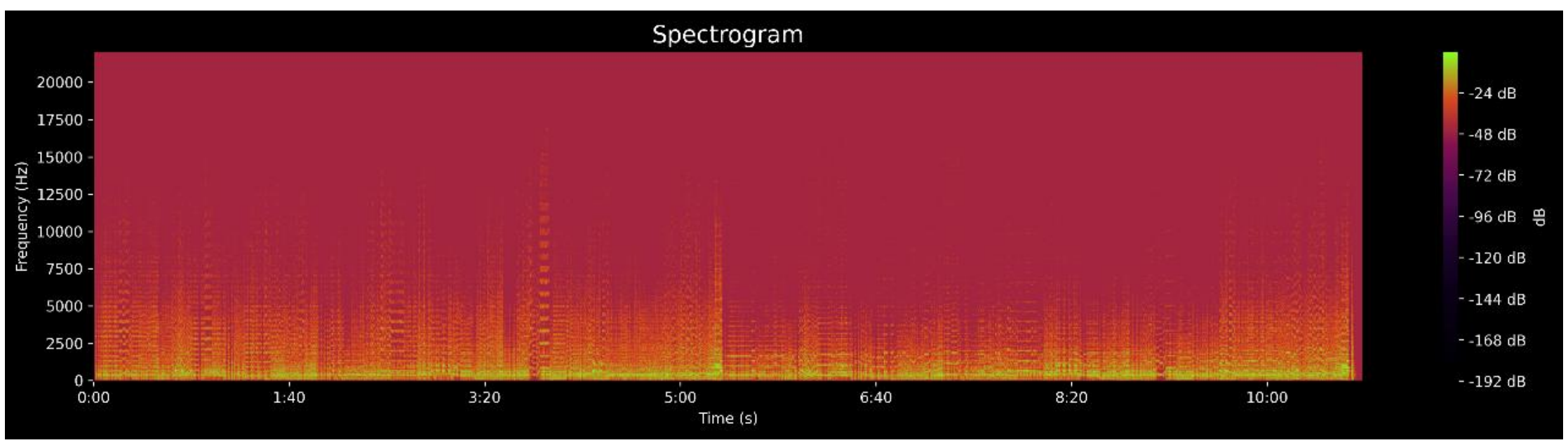
Spectrogram
Frequency (Hz)
0
2500
5000
7500
10000
12500
15000
17500
20000
Time (s)
0:00
1:40
3:20
5:00
6:40
8:20
10:00
dB
-24 dB
-48 dB
-72 dB
-96 dB
-120 dB
-144 dB
-168 dB
-192 dB

(D)

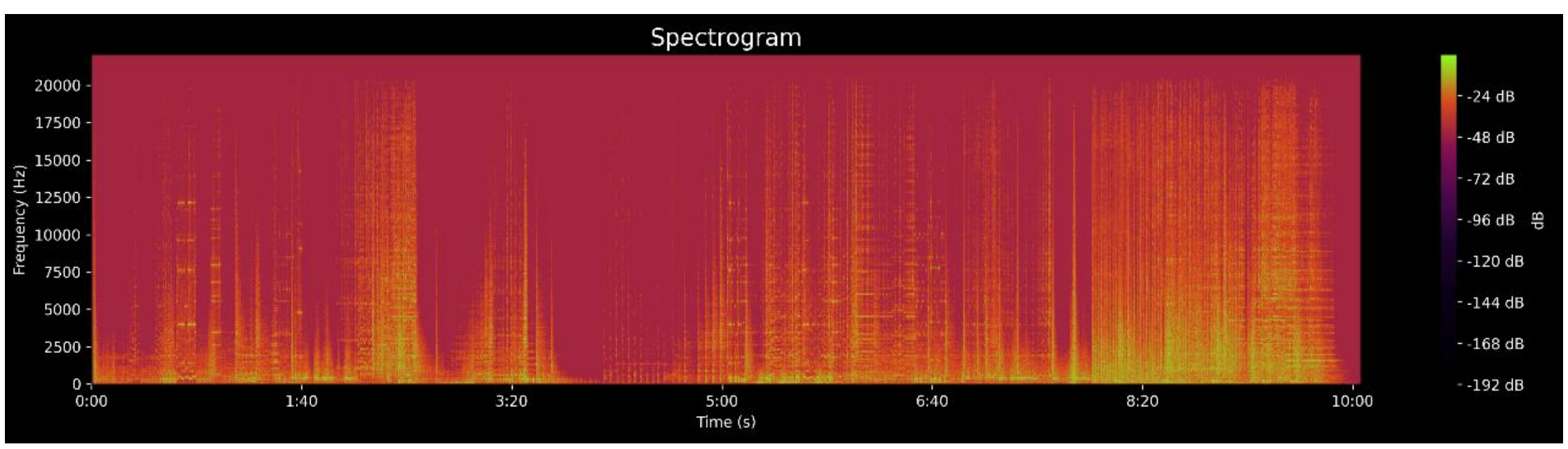
Spectrogram
Frequency (Hz)
0
2500
5000
7500
10000
12500
15000
17500
20000
Time (s)
0:00
1:40
3:20
5:00
6:40
8:20
10:00
dB
-24 dB
-48 dB
-72 dB
-96 dB
-120 dB
-144 dB
-168 dB
-192 dB

(E)

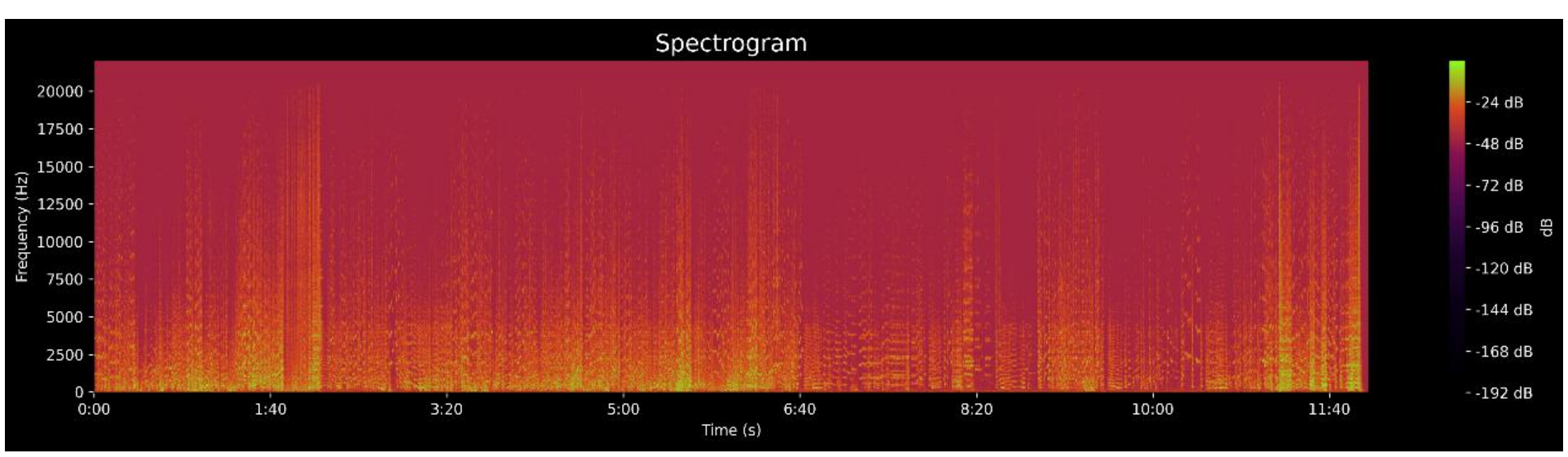
Spectrogram
Frequency (Hz)
0
2500
5000
7500
10000
12500
15000
17500
20000
Time (s)
0:00
1:40
3:20
5:00
6:40
8:20
10:00
11:40
dB
-24 dB
-48 dB
-72 dB
-96 dB
-120 dB
-144 dB
-168 dB
-192 dB

Figure 3. Music spectrograms for fast tempo, irregular rhythm, and atonal harmony music modality for composers **(A)** Callum, **(B)** Drew, **(C)** Jasmine, pre-composed music **(D)** John Cage **(D)**, and **(E)** Arnold Schoenberg. Frequency measurement (Hz) was recorded using height of peaks in each spectrogram. The color scale represents amplitude levels in dB.

Supplementary Tables

Table 1. Microsomal matrix partial-validation data for the liquid chromatography-tandem mass spectrometry (LC-MS/MS) assay for MPAG and AcMPAG (N=5 replicates)

Analytes	Concentration ng/mL		Accuracy, %	CV, %
MPAG	HQC	3200	96.21	10.52
	MQC	1600	92.39	12.82
	LQC	12.96	105.19	14.38
	LLOQ	4.67	99.58	15.99
AcMPAG	HQC	200	110.05	2.11
	MQC	100	97.37	3.07
	LQC	12.96	106.74	13.26
	LLOQ	4.67	113.25	17.49

AcMPAG: mycophenolic acid acyl glucuronide, CV%: coefficient of variation; HQC: high quality control concentration; LQC: low quality control concentration; LLOQ: lower limit of quantification; MPAG: mycophenolic acid glucuronide; MQC: middle quality control concentration.

Table 2A. Effects of tempo and rhythm on MPA glucuronide formation rates for individual rats (for manuscript figure 1A-B)

Group	MPAG formation reaction rates (nmol/mg/min) (Figure 1A)								Mean	SEM	p-value (vs control)
	F1	F2	F3	F4	M1	M2	M3	M4			
Control	0.0246	0.0356	0.0410	0.0238	0.0229	0.0221	0.0276	0.0306	0.0285	0.0024	
FT RR	0.0326	0.0240	0.0273	0.0219	0.0344	0.0191	0.0301	0.0266	0.0270	0.0019	0.9859
ST RR	0.0187	0.0227	0.0248	0.0287	0.0277	0.0246	0.0242	0.0452	0.0271	0.0028	0.9884
ST IR	0.0248	0.0209	0.0195	0.0257	0.0186	0.0233	0.0319	0.0258	0.0238	0.0015	0.5227
FT IR	0.0226	0.0321	0.0225	0.0229	0.0301	0.0262	0.0252	0.0354	0.0271	0.0017	0.9896
**Group**	**AcMPAG formation reaction rates (nmol/mg/min) (Figure 1B)**								**Mean**	**SEM**	**p-value (vs control)**
	**F1**	**F2**	**F3**	**F4**	**M1**	**M2**	**M3**	**M4**			
Control	0.0062	0.0095	0.0084	0.0052	0.0039	0.0036	0.0038	0.0055	0.0058	0.0008	
FT RR	0.0063	0.0052	0.0062	0.0060	0.0051	0.0038	0.0045	0.0037	0.0051	0.0004	>0.9999
ST RR	0.0031	0.0058	0.0048	0.0042	0.0027	0.0037	0.0042	0.0045	0.0041	0.0003	0.9531
ST IR	0.0052	0.0048	0.0054	0.0063	0.0042	0.0031	0.0025	0.0034	0.0044	0.0005	>0.9999
FT IR	0.0045	0.0030	0.0028	0.0037	0.0036	0.0030	0.0030	0.0030	0.0033	0.0002	0.0144

AcMPAG: mycophenolic acid acyl glucuronide, F: female, FT: fast tempo, IR: irregular rhythm, M: male, MPA: mycophenolic acid, MPAG: mycophenolic acid glucuronide, RR: regular rhythm, SEM: standard error of the mean, ST: slow tempo.

p-values for FT RR vs. ST RR, FT RR vs. ST IR, FT RR vs. FT IR, ST RR vs. ST IR, ST RR vs. FT IR, and ST IR vs. FT IR for MPAG formation were >0.9999, 0.8226, >0.9999, 0.8098, >0.9999 and 0.8027; for AcMPAG formation were >0.9999, >0.9999, 0.0193, >0.9999, >0.9999, and 0.5427 respectively.

Table 2B. Effects of tempo and rhythm on MPA glucuronide formation rates for individual female rats (for manuscript figure 1 C-D)

Group	MPAG formation reaction rates (nmol/mg/min) (Figure 1C)				Mean	SEM	p-value (vs control)
	F1	F2	F3	F4			
Control	0.0246	0.0356	0.0410	0.0238	0.0312	0.0042	
FT RR	0.0326	0.0240	0.0273	0.0219	0.0265	0.0023	0.7096
ST RR	0.0187	0.0227	0.0248	0.0287	0.0237	0.0021	0.3147
ST IR	0.0248	0.0209	0.0195	0.0257	0.0227	0.0015	0.21
FT IR	0.0226	0.0321	0.0225	0.0229	0.0250	0.0024	0.4897
**Group**	**AcMPAG formation reaction rates (nmol/mg/min) (Figure 1D)**				**Mean**	**SEM**	**p-value (vs control)**
	**F1**	**F2**	**F3**	**F4**			
Control	0.0062	0.0095	0.0084	0.0052	0.0073	0.0010	
FT RR	0.0063	0.0052	0.0062	0.0060	0.0059	0.0003	0.432
ST RR	0.0031	0.0058	0.0048	0.0042	0.0045	0.0006	0.0183
ST IR	0.0052	0.0048	0.0054	0.0063	0.0054	0.0003	0.1623
FT IR	0.0045	0.0030	0.0028	0.0037	0.0035	0.0004	0.0017

AcMPAG: mycophenolic acid acyl glucuronide, F: female, FT: fast tempo, IR: irregular rhythm, MPA: mycophenolic acid, MPAG: mycophenolic acid glucuronide, RR: regular rhythm, SEM: standard error of the mean, ST: slow tempo.

p-values for FT RR vs. ST RR, FT RR vs. ST IR, FT RR vs. FT IR, ST RR vs. ST IR, ST RR vs. FT IR, and ST IR vs. FT IR for MPAG formation were 0.9487, 0.8561, 0.9952, 0.9987, 0.9967, and 0.9709; for AcMPAG formation were 0.3782, 0.9605, 0.0485, 0.7553, 0.727, and 0.1587 respectively.

Table 2C. Effects of tempo and rhythm on MPA glucuronide formation rates for individual male rats (for manuscript figure 1 E-F)

Group	MPAG formation reaction rates (nmol/mg/min) (Figure 1E)				Mean	SEM	p-value (vs control)
	M1	M2	M3	M4			
Control	0.0229	0.0221	0.0276	0.0306	0.0258	0.0020	
FT RR	0.0344	0.0191	0.0301	0.0266	0.0276	0.0032	0.9949
ST RR	0.0277	0.0246	0.0242	0.0452	0.0304	0.0050	0.8483
ST IR	0.0186	0.0233	0.0319	0.0258	0.0249	0.0028	0.9996
FT IR	0.0301	0.0262	0.0252	0.0354	0.0292	0.0023	0.9426
**Group**	**AcMPAG formation reaction rates (nmol/mg/min) (Figure 1F)**				**Mean**	**SEM**	**p-value (vs control)**
	**M1**	**M2**	**M3**	**M4**			
Control	0.0039	0.0036	0.0038	0.0055	0.0042	0.0005	
FT RR	0.0051	0.0038	0.0045	0.0037	0.0043	0.0003	0.9998
ST RR	0.0027	0.0037	0.0042	0.0045	0.0038	0.0004	0.915
ST IR	0.0042	0.0031	0.0025	0.0034	0.0033	0.0004	0.4308
FT IR	0.0036	0.0030	0.0030	0.0030	0.0031	0.0002	0.2756

AcMPAG: mycophenolic acid acyl glucuronide, FT: fast tempo, IR: irregular rhythm, M: male, MPA: mycophenolic acid, MPAG: mycophenolic acid glucuronide, RR: regular rhythm, SEM: standard error of the mean, ST: slow tempo.

p-values for FT RR vs. ST RR, FT RR vs. ST IR, FT RR vs. FT IR, ST RR vs. ST IR, ST RR vs. FT IR, and ST IR vs. FT IR for MPAG formation were 0.9688, 0.9764, 0.996, 0.7505, 0.9988, and 0.8775; for AcMPAG formation were 0.8502, 0.3466, 0.2139, 0.8839, 0.7239, and 0.9972 respectively.

Table 3A. Effects of fast tempo, irregular rhythm, with tonal or atonal harmony on MPA glucuronide formation rates in individual rats (for manuscript figure 2A-B)

Group	MPAG formation reaction rates (nmol/mg/min) (Figure 2A)								Mean	SEM	p-value (vs control)
	F1	F2	F3	F4	M1	M2	M3	M4			
Control	0.01542	0.02293	0.02622	0.01547	0.01574	0.01604	0.02126	0.01925	0.01904	0.0014	
FT IR TH	0.01292	0.01967	0.01496	0.01552	0.01521	0.01860	0.02204	0.01410	0.01663	0.0011	0.4394
FT IR AH	0.01885	0.02468	0.01870	0.01400	0.01175	0.02101	0.01507	0.01443	0.01731	0.0015	0.6489
**Group**	**AcMPAG formation reaction rates (nmol/mg/min) (Figure 2B)**								**Mean**	**SEM**	**p-value (vs control)**
	**F1**	**F2**	**F3**	**F4**	**M1**	**M2**	**M3**	**M4**			
Control	0.0038	0.0063	0.0061	0.0036	0.0026	0.0024	0.0027	0.0035	0.0039	0.0005	
FT IR TH	0.0053	0.0059	0.0036	0.0037	0.0013	0.0013	0.0021	0.0011	0.0030	0.0007	>0.9999
FT IR AH	0.0019	0.0018	0.0012	0.0008	0.0004	0.0006	0.0005	0.0007	0.0010	0.0002	0.0014

AcMPAG: mycophenolic acid acyl glucuronide, F: female, FT IR TH: fast tempo, irregular rhythm, and tonal harmony; FT IR AH: fast tempo, irregular rhythm, and atonal harmony, M: male, MPA: mycophenolic acid, MPAG: MPA glucuronide, SEM: standard error of the mean.
p-values for MPAG and AcMPAG formation for FT IR TH vs FT IR AH were 0.9342 and 0.0327 respectively.

Table 3B. Effects of fast tempo, irregular rhythm, with tonal or atonal harmony on MPA glucuronide formation rates in female rats (for manuscript figure 2 C-D)

Group	MPAG formation reaction rates (nmol/mg/min) (Figure 2C)				Mean	SEM	p-value (vs control)
	F1	F2	F3	F4			
Control	0.0154	0.0229	0.0262	0.0155	0.0200	0.0027	
FT IR TH	0.0129	0.0197	0.0150	0.0155	0.0158	0.0014	0.3912
FT IR AH	0.0189	0.0247	0.0187	0.0140	0.0191	0.0022	0.9488
**Group**	**AcMPAG formation reaction rates (nmol/mg/min) (Figure 2D)**				**Mean**	**SEM**	**p-value (vs control)**
	**F1**	**F2**	**F3**	**F4**			
Control	0.0038	0.0063	0.0061	0.0036	0.0049	0.0007	
FT IR TH	0.0053	0.0059	0.0036	0.0037	0.0046	0.0006	0.9074
FT IR AH	0.0019	0.0018	0.0012	0.0008	0.0014	0.0003	0.0039

AcMPAG: mycophenolic acid acyl glucuronide, F: female, FT IR TH: fast tempo, irregular rhythm, and tonal harmony; FT IR AH: fast tempo, irregular rhythm, and atonal harmony, MPA: mycophenolic acid, MPAG: MPA glucuronide, SEM: standard error of the mean.
p-values for MPAG and AcMPAG formation for FT IR TH vs FT IR AH were 0.5554 and 0.0071 respectively.

Table 3C. Effects of fast tempo, irregular rhythm, with tonal or atonal harmony on MPA glucuronide formation rates in male rats (for manuscript figure 2 E-F)

Group	MPAG formation reaction rates (nmol/mg/min) (Figure 2E)				Mean	SEM	p-value (vs control)
	M1	M2	M3	M4			
Control	0.0157	0.0160	0.0213	0.0193	0.0181	0.0013	
FT IR TH	0.0152	0.0186	0.0220	0.0141	0.0175	0.0018	0.9687
FT IR AH	0.0117	0.0210	0.0151	0.0144	0.0156	0.0020	0.5744
**Group**	**AcMPAG formation reaction rates (nmol/mg/min) (Figure 2F)**				**Mean**	**SEM**	**p-value (vs control)**
	**M1**	**M2**	**M3**	**M4**			
Control	0.0026	0.0024	0.0027	0.0035	0.0028	0.0003	
FT IR TH	0.0013	0.0013	0.0021	0.0011	0.0015	0.0002	0.0019
FT IR AH	0.0004	0.0006	0.0005	0.0007	0.0006	0.0001	0.0001

AcMPAG: mycophenolic acid acyl glucuronide, FT IR TH: fast tempo, irregular rhythm, and tonal harmony; FT IR AH: fast tempo, irregular rhythm, and atonal harmony, M: male, MPA: mycophenolic acid, MPAG: MPA glucuronide, SEM: standard error of the mean.
p-values for MPAG and AcMPAG formation for FT IR TH vs FT IR AH were 0.7151 and 0.0235 respectively.

Table 4A. Comparison between three different composers of fast tempo, irregular rhythm, with atonal harmony music on MPA glucuronide formation rates in individual rats (for manuscript figure 4 A-B)

**Group**	**MPAG formation reaction rates (nmol/mg/min) (Figure 4A)**								**Mean**	**SEM**	**p-value (vs control)**
	**F1**	**F2**	**F3**	**F4**	**M1**	**M2**	**M3**	**M4**			
Control	0.0140	0.0177	0.0296	0.0166	0.0206	0.0207	0.0228	0.0266	0.0211	0.0018	
Callum	0.0151	0.0224	0.0145	0.0145	0.0128	0.0204	0.0192	0.0177	0.0171	0.0012	0.3655
Jasmine	0.0170	0.0128	0.0136	0.0141	0.0212	0.0214	0.0189	0.0193	0.0173	0.0012	0.4141
Drew	0.0093	0.0202	0.0176	0.0223	0.0154	0.0111	0.0301	0.0165	0.0178	0.0023	0.5446
**Group**	**AcMPAG formation reaction rates (nmol/mg/min) (Figure 4B)**								**Mean**	**SEM**	**p-value (vs control)**
	**F1**	**F2**	**F3**	**F4**	**M1**	**M2**	**M3**	**M4**			
Control	0.0028	0.0036	0.0052	0.0034	0.0037	0.0027	0.0034	0.0046	0.0037	0.0003	
Callum	0.0011	0.0013	0.0012	0.0011	0.0008	0.0007	0.0008	0.0010	0.0010	0.0001	0.0029
Jasmine	0.0008	0.0011	0.0009	0.0011	0.0007	0.0008	0.0005	0.0007	0.0008	0.0001	<0.0001
Drew	0.0016	0.0032	0.0041	0.0035	0.0013	0.0015	0.0017	0.0016	0.0023	0.0004	>0.9999

AcMPAG: mycophenolic acid acyl glucuronide, F: female, M: male, MPA: mycophenolic acid, MPAG: mycophenolic acid glucuronide, SEM: standard error of the mean.
p-values for MPAG formation for Callum vs. Jasmine, Callum vs. Drew, and Jasmine vs. Drew were 0.9997, 0.9892, and 0.996 respectively. p-values for AcMPAG formation for Callum vs. Jasmine, Callum vs. Drew, and Jasmine vs. Drew were >0.9999, 0.0988, and 0.0063 respectively.

Table 4B. Comparison between three different composers of fast tempo, irregular rhythm, with atonal harmony music on MPA glucuronide formation rates in female rats (for manuscript figure 4 C-D)

**Group**	**MPAG formation reaction rates (nmol/mg/min) (Figure 4C)**				**Mean**	**SEM**	**p-value (vs control)**
	**F1**	**F2**	**F3**	**F4**			
Control	0.0140	0.0177	0.0296	0.0166	0.0195	0.0035	
Callum	0.0151	0.0224	0.0145	0.0145	0.0166	0.0019	0.8485
Jasmine	0.0170	0.0128	0.0136	0.0141	0.0144	0.0009	0.4915
Drew	0.0093	0.0202	0.0176	0.0223	0.0174	0.0028	0.9297
**Group**	**AcMPAG formation reaction rates (nmol/mg/min) (Figure 4D)**				**Mean**	**SEM**	**p-value (vs control)**
	**F1**	**F2**	**F3**	**F4**			
Control	0.0028	0.0036	0.0052	0.0034	0.0038	0.0005	
Callum	0.0011	0.0013	0.0012	0.0011	0.0012	0.0000	0.0018
Jasmine	0.0008	0.0011	0.0009	0.0011	0.0010	0.0001	0.001
Drew	0.0016	0.0032	0.0041	0.0035	0.0031	0.0005	0.5817

AcMPAG: mycophenolic acid acyl glucuronide, F: female, MPA: mycophenolic acid, MPAG: mycophenolic acid glucuronide, SEM: standard error of the mean.
p-values for MPAG formation for Callum vs. Jasmine, Callum vs. Drew, and Jasmine vs. Drew were 0.9151, 0.9966, and 0.8277 respectively. p-values for AcMPAG formation for Callum vs. Jasmine, Callum vs. Drew, and Jasmine vs. Drew were 0.9789, 0.0168, and 0.0085 respectively.

Table 4C. Comparison between three different composers of fast tempo, irregular rhythm, with atonal harmony music on MPA glucuronide formation rates in male rats (for manuscript figure 4 E-F)

**Group**	**MPAG formation reaction rates (nmol/mg/min) (Figure 4E)**				**Mean**	**SEM**	**p-value (vs control)**
	**M1**	**M2**	**M3**	**M4**			
Control	0.0206	0.0207	0.0228	0.0266	0.0227	0.0014	
Callum	0.0128	0.0204	0.0192	0.0177	0.0175	0.0016	0.4393
Jasmine	0.0212	0.0214	0.0189	0.0193	0.0202	0.0006	0.8809
Drew	0.0154	0.0111	0.0301	0.0165	0.0183	0.0041	0.5695
**Group**	**AcMPAG formation reaction rates (nmol/mg/min) (Figure 4F)**				**Mean**	**SEM**	**p-value (vs control)**
	**M1**	**M2**	**M3**	**M4**			
Control	0.0037	0.0027	0.0034	0.0046	0.0036	0.0004	
Callum	0.0008	0.0007	0.0008	0.0010	0.0008	0.0001	0.0694
Jasmine	0.0007	0.0008	0.0005	0.0007	0.0007	0.0001	0.0038
Drew	0.0013	0.0015	0.0017	0.0016	0.0015	0.0001	>0.9999

AcMPAG: mycophenolic acid acyl glucuronide, M: male, MPA: mycophenolic acid, MPAG: mycophenolic acid glucuronide, SEM: standard error of the mean.

p-values for MPAG formation for Callum vs. Jasmine, Callum vs. Drew, and Jasmine vs. Drew were 0.8456, 0.9952, and 0.9354 respectively. p-values for AcMPAG formation for Callum vs. Jasmine, Callum vs. Drew, and Jasmine vs. Drew were >0.9999, >0.9999, and 0.1554 respectively.

Table 5A. Effects of published, pre-composed music with fast tempo, irregular rhythm, with atonal harmony music on MPA glucuronides formation rates in individual rats (for manuscript figure 5A-B)

Group	MPAG formation reaction rates (nmol/mg/min) (Figure 5A)								Mean	SEM	p-value (vs control)
	F1	F2	F3	F4	M1	M2	M3	M4			
Control	0.0140	0.0177	0.0296	0.0166	0.0206	0.0207	0.0228	0.0266	0.0211	0.0018	
John Cage	0.0140	0.0120	0.0197	0.0232	0.0248	0.0242	0.0168	0.0208	0.0194	0.0017	0.7458
Arnold Schoenberg	0.0147	*a*	0.0168	0.0159	0.0190	0.0229	0.0191	0.0165	0.0179	0.0010	0.3612
**Group**	**AcMPAG formation reaction rates (nmol/mg/min) (Figure 5B)**								**Mean**	**SEM**	**p-value (vs control)**
	**F1**	**F2**	**F3**	**F4**	**M1**	**M2**	**M3**	**M4**			
Control	0.0028	0.0036	0.0052	0.0034	0.0037	0.0027	0.0034	0.0046	0.0037	0.0003	
John Cage	0.0025	0.0026	0.0035	0.0046	0.0039	0.0027	0.0026	0.0024	0.0031	0.0003	0.3327
Arnold Schoenberg	0.0033	*a*	0.0037	0.0027	0.0021	0.0022	0.0017	0.0019	0.0025	0.0003	0.0297

AcMPAG: mycophenolic acid acyl glucuronide, F: female, M: male, MPA: mycophenolic acid, MPAG: mycophenolic acid glucuronide, SEM: standard error of the mean.
p-values for MPAG and AcMPAG formation for John Cage vs Arnold Schoenberg were 0.7738 and 0.3723 respectively.
a: Female 2 from Arnold Schoenberg's music exposed group was deceased and no data available.

Table 5B. Effects of published, pre-composed music with fast tempo, irregular rhythm, with atonal harmony music on MPA glucuronides formation rates in female rats (for manuscript figure 5 C-D)

Group	MPAG formation reaction rates (nmol/mg/min) (Figure 5C)				Mean	SEM	p-value (vs control)
	F1	F2	F3	F4			
Control	0.0140	0.0177	0.0296	0.0166	0.0195	0.0035	
John Cage	0.0140	0.0120	0.0197	0.0232	0.0172	0.0026	0.8284
Arnold Schoenberg	0.0147	*a*	0.0168	0.0159	0.0158	0.0006	0.656
**Group**	**AcMPAG formation reaction rates (nmol/mg/min) (Figure 5D)**				**Mean**	**SEM**	**p-value (vs control)**
	**F1**	**F2**	**F3**	**F4**			
Control	0.0028	0.0036	0.0052	0.0034	0.0038	0.0005	
John Cage	0.0025	0.0026	0.0035	0.0046	0.0033	0.0005	0.7417
Arnold Schoenberg	0.0033	*a*	0.0037	0.0027	0.0033	0.0003	0.7355

AcMPAG: mycophenolic acid acyl glucuronide, F: female, MPA: mycophenolic acid, MPAG: mycophenolic acid glucuronide, SEM: standard error of the mean.
p-values for MPAG and AcMPAG formation for John Cage vs Arnold Schoenberg were 0.9348 and 0.9976 respectively.
a: Female 2 from Arnold Schoenberg's music exposed group was deceased and no data available.

Table 5C. Effects of published, pre-composed music with fast tempo, irregular rhythm, with atonal harmony music on MPA glucuronides formation rates in male rats (for manuscript figure 5 E-F)

**Group**	**MPAG formation reaction rates (nmol/mg/min) (Figure 5E)**				**Mean**	**SEM**	**p-value (vs control)**
	**M1**	**M2**	**M3**	**M4**			
Control	0.0206	0.0207	0.0228	0.0266	0.0227	0.0014	
John Cage	0.0248	0.0242	0.0168	0.0208	0.0216	0.0018	0.8853
Arnold Schoenberg	0.0190	0.0229	0.0191	0.0165	0.0194	0.0013	0.3365
**Group**	**AcMPAG formation reaction rates (nmol/mg/min) (Figure 5F)**				**Mean**	**SEM**	**p-value (vs control)**
	**M1**	**M2**	**M3**	**M4**			
Control	0.0037	0.0027	0.0034	0.0046	0.0036	0.0004	
John Cage	0.0039	0.0027	0.0026	0.0024	0.0029	0.0003	0.2901
Arnold Schoenberg	0.0021	0.0022	0.0017	0.0019	0.0020	0.0001	0.0119

AcMPAG: mycophenolic acid acyl glucuronide, M: male, MPA: mycophenolic acid, MPAG: mycophenolic acid glucuronide, SEM: standard error of the mean.
p-values for MPAG and AcMPAG formation for John Cage vs Arnold Schoenberg were 0.5807 and 0.1426 respectively.
